# Visual perception of emotion cues in dogs: a critical review of methodologies

**DOI:** 10.1007/s10071-023-01762-5

**Published:** 2023-03-04

**Authors:** Catia Correia-Caeiro, Kun Guo, Daniel S. Mills

**Affiliations:** 1 School of Psychology, University of Lincoln, Brayford Pool, Lincoln, LN6 7TS UK; 2 Department of Life Sciences, University of Lincoln, Lincoln, LN6 7DL UK; 3 Primate Research Institute, Kyoto University, Inuyama, 484-8506 Japan; 4 Center for the Evolutionary Origins of Human Behavior, Kyoto University, Inuyama, 484-8506 Japan

**Keywords:** Emotion cues, Visual perception, Facial expressions, Bodily expressions, Human–dog relationship, Methodology

## Abstract

Comparative studies of human–dog cognition have grown exponentially since the 2000’s, but the focus on how dogs look at us (as well as other dogs) as social partners is a more recent phenomenon despite its importance to human–dog interactions. Here, we briefly summarise the current state of research in visual perception of emotion cues in dogs and why this area is important; we then critically review its most commonly used methods, by discussing conceptual and methodological challenges and associated limitations in depth; finally, we suggest some possible solutions and recommend best practice for future research. Typically, most studies in this field have concentrated on facial emotional cues, with full body information rarely considered. There are many challenges in the way studies are conceptually designed (e.g., use of non-naturalistic stimuli) and the way researchers incorporate biases (e.g., anthropomorphism) into experimental designs, which may lead to problematic conclusions. However, technological and scientific advances offer the opportunity to gather much more valid, objective, and systematic data in this rapidly expanding field of study. Solving conceptual and methodological challenges in the field of emotion perception research in dogs will not only be beneficial in improving research in dog–human interactions, but also within the comparative psychology area, in which dogs are an important model species to study evolutionary processes.

**Supplementary Information:**

The online version contains supplementary material available at 10.1007/s10071-023-01762-5.

## Introduction

**Fig. 1:**
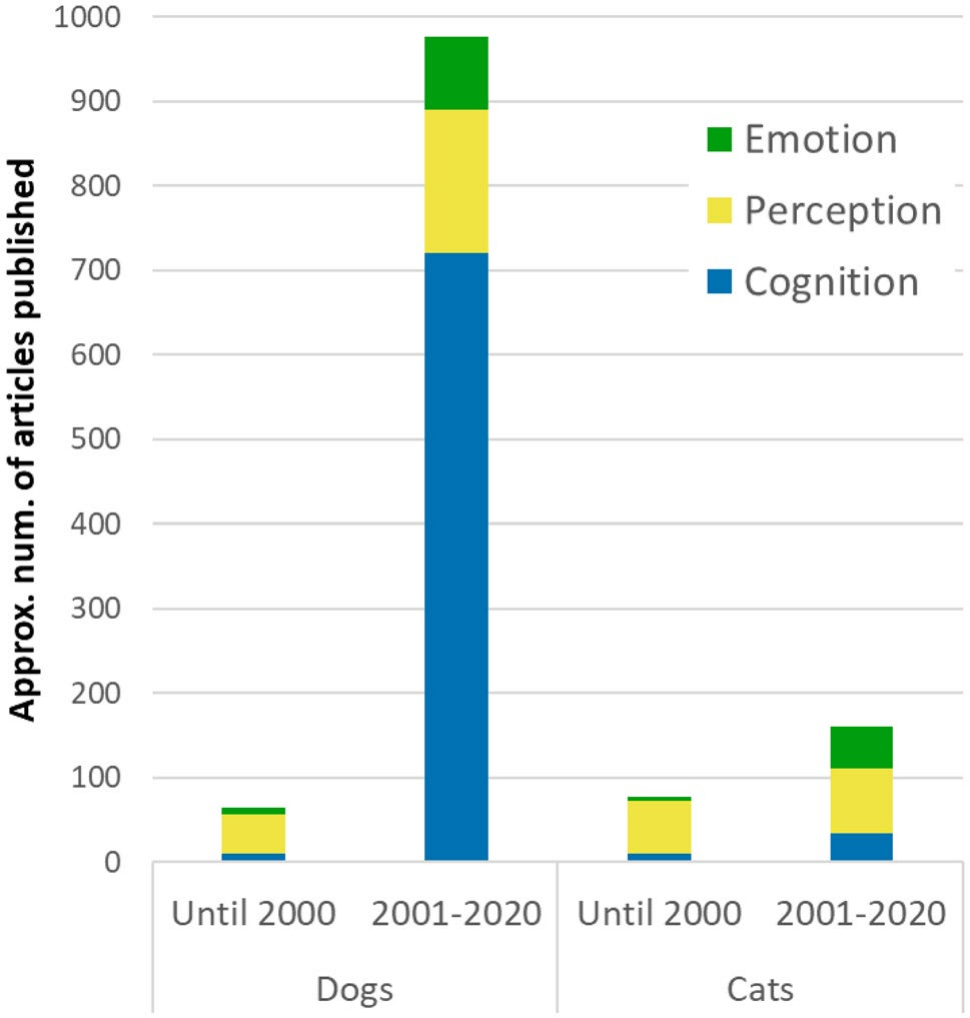
Comparison of articles published until 2000 and from 2001 till 2020 available on GoogleScholar, searched using the keywords “dog cognition”, “dog perception”, or “dog emotion”. The same search using the terms “cat cognition”, “cat perception”, or “cat emotion” was used for comparison purposes in the same periods. The explosion of studies in these areas is particularly evident since the turn of the millennium for dogs: up to the year 2000, GoogleScholar displays only 10 results when searching, for example, for “dog cognition”, but in the next 20 years period (2001–2020) 720 results appear; “dog perception” returns 47 results pre-2000 and 170 studies since, while “dog emotion” returns 7 results pre-2000 and 86 results since. This represents a 72, 3.6, and 12.2 times increase for these research topics in dogs, respectively compared with only 3.4, 1.2, and tenfold increase for cats

Several reviews have been published recently on dog cognition (Arden et al. 2016; Bensky et al. 2013; Kubinyi et al. 2007; Lea and Osthaus 2018; Miklósi and Kubinyi 2016; Wynne 2016), visual abilities (Barber et al. 2020; Byosiere et al. 2018; Miller and Murphy 1995), and dog–human communication (Siniscalchi et al. 2018a, b), including attention to pointing gestures (Kaminski and Nitzschner 2013) and faces (Huber 2016). Another review (Kujala 2017), followed by a thread of invited commentaries, explored the questions of if and how dogs may experience emotion, but no review has so far focused on the issue of perception of emotion cues, and more importantly on the methodologies used to study this topic. An increase in studies (Fig. 1) has been changing the status of the domestic dog in biological research, from inadequate/irrelevant for “real biology” due to its domestication, to an ideal model species (Cooper et al. 2003; Miklosi 2014; Topal et al. 2009) for understanding a range of phenomena, from explanations of their uniqueness (Miklosi 2014; Prato-Previde and Marshall-Pescini 2014) to the evolution of communication and emotion in humans and non-human animals (Andics et al. 2014; Gruber and Bekoff 2017; Hare 2007). Given this increased scientific interest in this field, an early critical appraisal of concepts and methodologies is timely for future research.

Here, we have used a critical review methodology (Grant and Booth 2009) to briefly summarise the most salient publications in this area, and to critically evaluate concepts and methodologies commonly applied, as well as what can be learned from them. We structured this critique to cover the following key questions in studying the perception of emotion cues in dogs:

1. What is the nature of emotion and its perceptual processes?
2. Why is the dog a good model species?
3. What is known about the perception of emotion cues in dogs?
4. What methodologies have been used to study the perception of emotion cues in dogs?
5. What are the limitations and challenges of studying the perception of emotion cues in dogs?
6. What are the scientific and practical considerations regarding the methodologies currently used when studying the perception of emotion cues in dogs?

Given that the perception of emotion cues is central to understanding social interactions in individuals, this review fills an important knowledge gap in the wider fields of emotion and cognition in dogs. We do not however address other topics such as the philosophical debate concerning what an emotion is nor other perceptual modalities, but we briefly summarise in the next section and in the Supplementary Text S1 the wider debate of emotion nature and function, and justify our focus on visual cues, respectively. We also do not intend to extensively review the evolutionary processes of dogs as a domestic species nor what is known about dogs' perception of emotion, but we give a brief summary of both topics in Sects. “Why is the dog a good model for research on the perception of emotion cues?” and “What is known about how dogs visually perceive emotion cues?” for a better understanding of the methodological critique. We conclude this review by briefly describing the methodologies used in this area and discussing its limitations and future considerations in Sects. “What methodologies have been used to assess the perception of emotion cues in dogs?” – “What are the limitations and challenges to investigate the perception of emotion cues in dogs?”, respectively.

## A brief summary of the nature of emotion and its perceptual processes

Emotion processes are thought to have evolved to allow individuals to avoid harm/punishment and seek valuable resources/rewards (Dawkins 2000; Duncan 2006; Paul et al. 2005; Rolls 2005). Emotions have been defined as short-lived internal states occurring in response to external or internal stimuli that are perceived to have a specific value to the individual (emotionally-competent stimuli), and produce both internal and external changes, including cognitive appraisal, physiological activation, motor expression and behavioural tendency (Scherer 2005). For example, if an aversive stimulus is identified, an array of internal responses (e.g., amygdala activation and release of CRF, Adolphs 2013; Panksepp 2011; increase in heart rate, LeDoux 2003; Thayer and Lane 2009) is usually accompanied by certain behavioural tendencies (e.g., flight) and expressive/communicative components (e.g., fearful face, Chevalier-Skolnikoff 1973; Darwin 1896; Leopold and Rhodes 2010). The external responses (i.e., emotion cues) are particularly important in social interactions, as they can be perceived and processed by other individuals present in the same environment (i.e., receivers). Even if signals evolve for the benefit of the senders and not the receivers, receivers still have the potential to use these as cues (i.e., any stimulus that an individual can detect and learn to use: Saleh et al. 2007) as valuable information to improve navigation of their social and physical environment (e.g., a fearful face in the sender might indicate an environmental danger). Hence, perception of emotion cues is critical for survival and increases fitness, but it is not a simple or straightforward task to accomplish due to differences in how emotions are activated and cues produced by the senders. These differences create a population of emotionally distinct individuals, who may not produce similar emotion cues in terms of type or intensity to certain stimuli (Anderson and Adolphs 2014). For example, more fearful individuals might produce cues related to flight (e.g., fearful facial expressions), freeze (e.g., absence of response or neutral face) or fight (e.g., angry facial expression) situations, which subsequently can have different outcomes impacting fitness and survival of both the sender and the receiver.

Whilst *emotions* are internal states and arise from multi-component complex biological and perceptual processes (and thus are subjective and hard to measure as a single concept), *emotion cues* are variably present on a sender, may be observable by a receiver, and belong to distinct modalities (and thus can be objectively quantified). Emotion cues are one of the ways of communicating between individuals which have not evolved to function as a signal (for distinction between cue and signal, see Freeberg et al. 2021). However, although we use the term “emotion cues” as described above, these cues do not contain emotion per se, i.e., facial expressions, body postures, vocalisations, etc., are not inherently an emotion and can be used independently from a particular emotion state. For example, in humans, a smile may be displayed when the individual is in a positive state or when the individual is simply greeting someone (see below for more on the multiple functions of facial expressions).

The biologically-based definition of emotion displays we use here is thus in line with the Basic Emotion Theory (BET, reviewed for example in Tracy and Randles 2011), in which it is agreed by different researchers that an internal state fits the criteria for basic emotion if (1) it is discrete, (2) presents fixed neural (subcortical) and behavioural correlates (i.e., the emotion cues), (3) has a fixed feeling or motivational response, and importantly, (4) it can be generalised across species (but not necessarily). Nonetheless, we recognise that there are opposing theories of emotion cues production, in particular regarding facial displays (e.g., Behavioral Ecology View: BECV proposes facial expressions to be disassociated from internal states, lacking fixed appearance changes or meanings, and instead act as “social tools”: Crivelli and Fridlund 2018), with growing debates on definitions and/or functions of both emotions and emotion cues (Barrett 2006; Crivelli and Fridlund 2019; Damasio 2003; Izard 2007; Jack et al. 2012a, b; Jack et al. 2014; Jack and Schyns 2015; Keltner et al. 2019; LeDoux 2015; Seyfarth and Cheney 2003), as well as questions on the universality of emotion cues (Chen and Jack 2017; Cowen et al. 2021; Cowen and Keltner 2017; Russell 1994; Volynets et al. 2020), or even its existence beyond a social construct (Barrett 2016). Nonetheless, these opposing views are not mutually exclusive, and they can be combined into a more nuanced view (Camerlink et al. 2018; Waller and Micheletta 2013), in which visual cues such as facial expressions can probably function both as emotional expressive or communicative cues. One of the classical examples in humans of this multi-function of facial expressions is probably the “smile” with its wide range of meanings and functions (Ekman and Friesen 1982), including for example the “felt” or Duchenne smile, which is a correlate of a positive internal state, or the “Pan-Am” smile (named due to its normative display by air crew greeting passengers) that is displayed as a greeting signal (uncorrelated to internal states). We support this more nuanced and complex view of emotions and emotion cues (encompassing BET and BECV), in which emotions are biologically well defined, but its measurable outputs can be both biological (e.g., facial expressions acting as emotion cues) or socially and evolutionary shaped (e.g., facial expressions acting as communication signals).

In any case, indubitably, investigating the perception (and by extension, production) of emotion cues in dogs may not only add to this debate by providing an evolutionary (e.g., which emotional processes are shared with humans and how/why they might have evolved?) and comparative perspective (e.g., what each species makes of emotion cues, are these emotion cues homologous or analogous in dogs?), but it might actually bypass a lot of the requirement issues (e.g., language, consciousness) that at the moment entangle the debates in emotion perception, production and experience.

Importantly, in this review we focus exclusively on what is known so far on how dogs are able to extract visual information from their environment (social and non-social) through their specialised visual system (e.g., Barber et al. 2020; Byosiere et al. 2018), and perceive emotion cues intra- and inter-specifically. Therefore, this review does not aim at (1) discussing theories of emotions, as this necessarily would include a much more extensive and broad work on experience of emotion as internal states, production of emotion and/or social cues, intentionality, flexibility, control over displays, etc.; (2) discussing the exclusivity of proximate (emotional) or ultimate (communicative) mechanisms of visual cues (for this, see for e.g., Waller et al. 2017); (3) reviewing human nor dog emotion-related experiences (e.g., feelings, moods, sensations) per se; (4) speculating on the meaning of cues as signals, as no study has yet empirically tested for this in dogs (e.g., by examining both sender and receiver simultaneously).

## Why is the dog a good model for research on the perception of emotion cues?

Despite being a species with a tremendous sense of smell, dogs seem to have a well-developed visual system (Barber et al. 2020; Byosiere et al. 2018) and a remarkable ability to visually read humans’ communication, emotions, and intentions (Arden et al. 2016; Huber 2016; Lea and Osthaus 2018; Reid 2009). The recent wealth of studies on the dog (Fig. 1) reveal this species is highly sensitive to visual social cues, particularly when it comes to human–dog communication. For example, dogs can take into account what other individuals can see (Kaminski et al. 2013; Savalli et al. 2013) or know (Catala et al. 2017; Maginnity and Grace 2014), follow human action to solve a task (Pongrácz et al. 2001), respond and adapt to human behaviour (Gácsi et al. 2013; Kaminski et al. 2017), understand human intentions and beliefs (Lonardo et al. 2021; Schünemann et al. 2021), and act to manipulate others' attention (Horowitz 2009). These abilities in perspective taking and attention sensitivity have been used to argue initially for a “rudimentary Theory of Mind” (ToM) in dogs (Horowitz 2011), and since then evidence on different aspects of ToM in dogs has been growing (Lea and Osthaus 2018; however, see (Wynne 2021) for an opposing view). Researchers have also found evidence of rapid facial mimicry (Palagi et al. 2015), contagious yawning (Joly-Mascheroni et al. 2008), pupillary (Axelsson and Fawcett 2020) and emotional contagion (Palagi et al. 2015), and empathy-like behaviour (Custance and Mayer 2012; Silva and Sousa 2011) in dogs. Dogs are also able to integrate cues from different modalities to extract emotion information (Albuquerque et al. 2016; Faragó et al. 2010) and show social referencing (Merola et al. 2013, 2014; Yong and Ruffman 2013). Although not all these abilities are exclusive of dogs (e.g., wolves also show perspective taking: Udell et al. 2011), and some aspects are still being debated (e.g., contagious yawning: Harr et al. 2009; O’Hara and Reeve 2011; Yoon and Tennie 2010) or empathy-like behaviour (Adriaense et al. 2020), taken together, these studies indicate that social and emotion cues are crucial for dogs' social interactions.

There is evidence that domestication has shaped dogs’ communicative and perceptual processes, where some differences have been noted between wolves and dogs (Gácsi et al. 2005, 2009; Johnston et al. 2017; Kubinyi et al. 2007; Lampe et al. 2017). On the other hand, similarities in the neurobiological basis for social abilities have been suggested between humans and dogs (Buttner 2016) as well as similarities in other social features, such as behavioural synchronisation, which potentially increases social cohesion and affiliation (Duranton and Gaunet 2018). More importantly, there is now wide evidence of interspecific emotion cues perception and recognition in different modalities (e.g., dog vocalisations, Pongrácz et al. 2006; human facial expressions, Albuquerque et al. 2016; Buttelmann and Tomasello 2013; Correia-Caeiro et al. 2020; Müller et al. 2015; Nagasawa et al. 2011; Pitteri et al. 2014a; Racca et al. 2012), lending further support to the proposed idea that co-evolution between humans and dogs within a shared environment may have occurred to some extent to create convergence in cognitive skills ((Hare 2007; Hare and Ferrans 2021; Hare and Tomasello 2005), but for further debate on this topic, see (Range and Marshall-Pescini 2022; Udell et al. 2010; Udell and Wynne 2008)).

The group size and complex dynamics of ancestral wolf-type populations may also have provided an important substrate for the evolution of the human–dog relationship. Typically, social species display complex social signals (Dobson 2009b, 2009a), which, according to the "Social brain hypothesis", also requires advanced cognition to navigate these more demanding social environments (Dunbar 1998; Whiten and Byrne 1988). This, coupled with the need to operate within a framework of rapid exchanges to prevent harm to either or both parties (Mills and Westgarth 2017), would favour the development of these abilities within both visual and acoustic sensory channels. The extraordinary proficiency of dogs in being able to read emotion cues in humans, might then be a key feature in their successful domestication and subsequent ubiquity in society in roles such as a companion, assistance and therapy animal as evidenced by their economic significance (Hall et al. 2016).

Taken together, the known wide set of perceptual skills, the co-evolutionary processes with humans, and the high sociability tendency make dogs a unique model species for studying the perception of emotion cues at both the intra and interspecific level.

## What is known about how dogs visually perceive emotion cues?

When dogs are exposed to an ambiguous/threatening situation, they gaze at humans to look for information about the situation and react according to the emotion cues expressed by their owners (Merola et al. 2012), with body movement and vocal intonation being enough to elicit social referencing (Salamon et al. 2020). Beyond negative situations, in the last decade or so, a wealth of research has focused on this question of what dogs perceive from human emotions cues. However, nearly all studies focused on how dogs perceive human facial expressions. This face bias might be due to an anthropocentric effect, since human faces are extremely important in conspecific social interactions (more so than the body or voice, Ekman et al. 1980). When humans interact with dogs they are very likely to display frequent facial cues, since dogs are seen as quasi-social partners by humans (Serpell 2009).

**Fig. 2:**
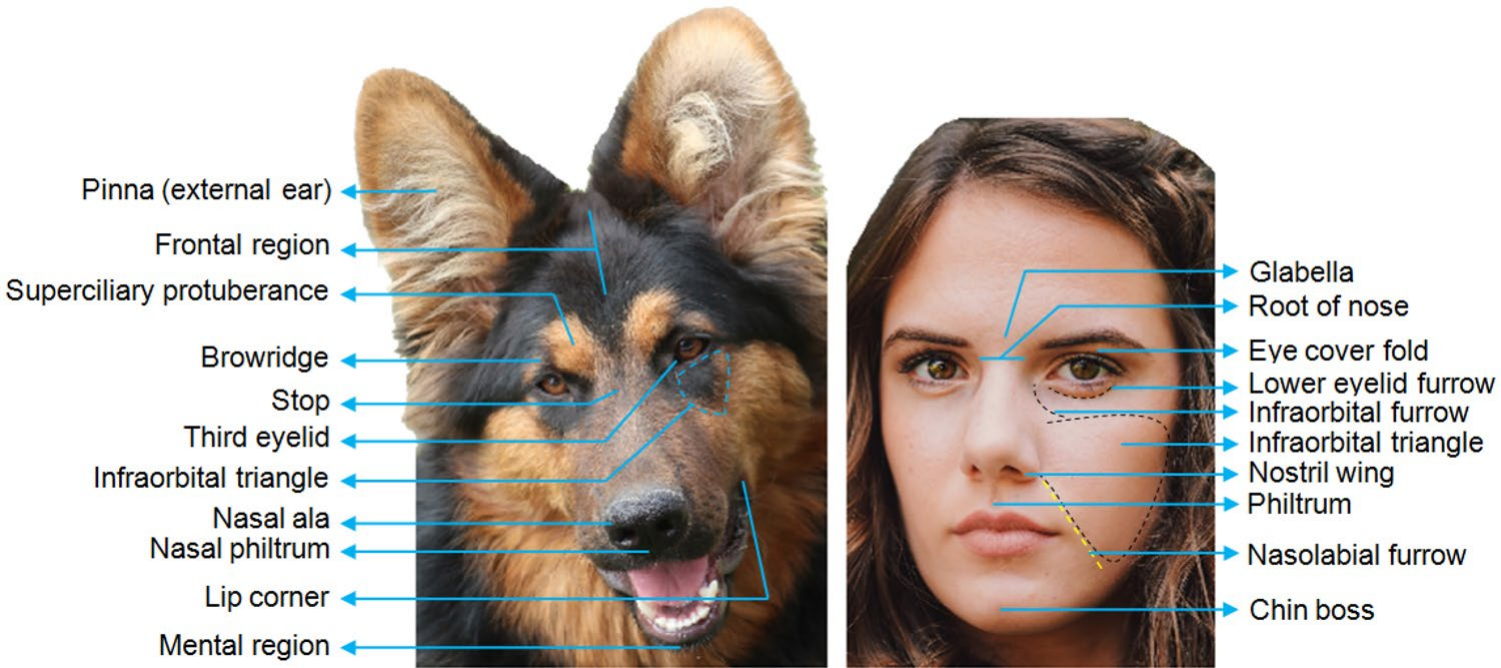
Facial landmarks in dogs and humans (adapted from DogFACS and HumanFACS, respectively: Ekman et al. 2002a; Waller et al. 2013). The FACS systems are anatomically-based, standardised and objective methods of facial coding that avoid subjective labelling (e.g., "smile"). The position of facial landmarks in both species is arranged differently due to the variation in anatomical features such as skull shape, fat deposits, and hair coverage. For example, dogs do not have a forehead or eyebrows (anatomical features unique to humans) and instead have a frontal region and browridges. Pictures by Mouse23 from Pixabay.com (2021) and by Natalie Heathcoat from Unsplash.com (2021), free for commercial use

Furthermore, a mechanism known as Face Based Emotion Recognition (FaBER) is suggested to be widespread in mammals with good visual acuity, including humans (Tate et al. 2006) and dogs (Lind et al. 2017), which may explain this face bias. However, despite humans being very facially expressive, and both humans and dogs being perhaps well-equipped to perceive each other's facial cues (due to FaBER), there is large variance in facial morphology (Fig. 2) and the display of emotion cues between species (Caeiro et al., 2017, Fig. 3). Thus, the next question is whether dogs can read and infer meaning from human facial expressions by overcoming the challenges in successfully decoding emotion signals across a species barrier.

**Fig. 3:**
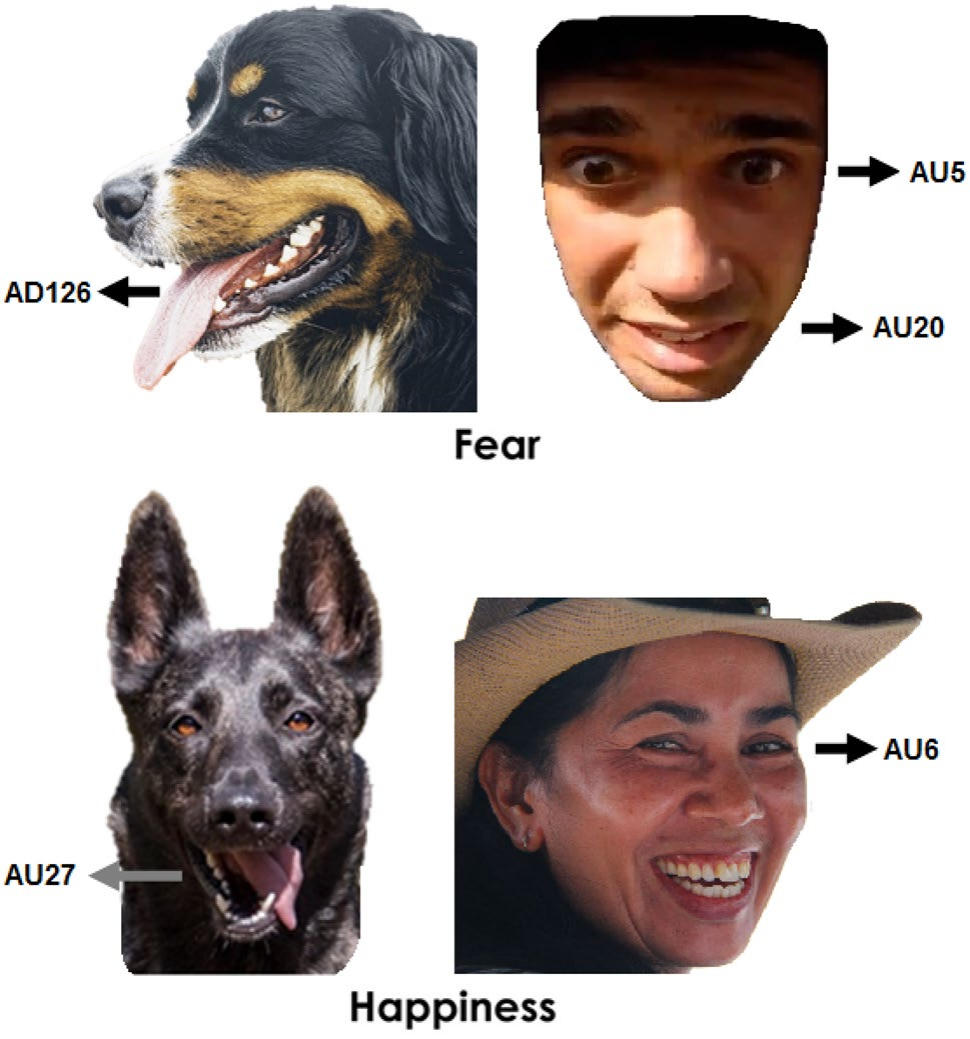
Example of differences between characteristic facial cues of emotion in a human and dog (Ekman et al. 2002a; Waller et al. 2013) in equivalent emotional contexts (Correia-Caeiro et al. 2017; Ekman et al. 1994). Fearful facial expressions in humans tend to include eyes wide open (AU5) and lip corners stretched horizontally (AU20) while dog fearful facial expressions tend to include panting (AD126). Happy facial expressions in humans tend to include the wrinkling around the eyes (AU6), while in dog happy facial expressions tend to include wide open mouths (AU27). AD: Action Descriptor, AU: Action Unit, AD126: Panting, AU5: Upper Lid Raise, AU6: Cheek Raise, AU20: Lip Stretch, AU27: Mouth Stretch. Dog images modified from Caeiro et al. (2017); Images by users Pexels and 2,843,603 from Pixabay.com (2021), free for commercial use, and by Sifis Kavroudakis from Youtube.com (2021)

In one study, dogs could discriminate human smiling faces from neutral faces (Nagasawa et al. 2011), but in another study (Buttelmann and Tomasello 2013) that included five breeds and two conditions (lab and open field), results were less clear: only one of the five breeds in the open field condition could discriminate happy from neutral faces. However, all breeds in both conditions could discriminate happy from disgusted faces (Buttelmann and Tomasello 2013), which might indicate some variation in breed ability and environment-dependent performance. Nonetheless, in these studies dogs showed expected differential reactions (approach/avoidance behaviours) when presented with joyful, angry, fearful and disgusted human faces compared with neutral face presentation. This suggests that the inconsistencies in both studies may be due to methodological differences in how dogs were tested, although it is also possible that opposite valences are easier for dogs to discriminate. Another study (Müller et al. 2015) in which dogs successfully discriminated opposite valences (happy vs angry) further examined how dogs were processing these facial cues. Here, dogs’ discrimination of facial expressions was shown to be based on configural cues, in which dogs might form associations based on previous experience of faces, between different regions of the face and its expression of emotion cues (Müller et al. 2015). Racca et al (2012) has also presented dog and human facial expressions with different valences (angry, neutral, and happy) to dogs, and observed a consistent Left Gaze Bias (LGB) for negative and neutral human facial expressions, but no bias for positive expressions. They argued that perhaps dogs interpret human neutral facial expressions as potentially negative, given their lack of clear signals to encourage approach. By contrast, there was a differential gaze asymmetry for dog faces based on their valence, with no gaze bias for neutral expressions but a LGB for negative expressions and a Right Gaze Bias (RGB) for positive expressions (Siniscalchi et al. 2010, 2013). This gaze asymmetry is possibly a reflection of brain lateralisation processes, also reflected in tail and head turning when facing or displaying emotion cues. This might indicate a more general mechanism for perception of emotion cues, in which left and right hemispheres are mainly involved in the processing of positive and negative emotions, respectively (Siniscalchi et al. 2008). Although it could be argued that the gaze bias in dogs might be related to approach/avoidance behaviour and is not necessarily correlated with emotion cue perception or emotion experience.

Several eye-tracking studies with dogs have provided further fine-grained information on how this species perceives visual cues (e.g., Barber et al. 2016; Park et al. 2019; Somppi et al. 2014; Téglás et al. 2012; Völter et al. 2020; Völter and Huber 2022; Williams et al. 2011). These studies have shown that, as with humans, dogs prefer to fixate more on the internal facial features (especially on the eyes) when viewing human and dog faces (Somppi et al. 2014) and process the composition formed by eyes, midface and mouth as a whole in facial expressions (Somppi et al. 2016). Furthermore, dogs seem to have a specific gazing pattern dependent on the facial expression they are looking at, which may be associated with their interpretation of the viewed expressions (Barber et al. 2016; Correia-Caeiro et al. 2020; Somppi et al. 2016). In one of these studies (Somppi et al. 2016), dogs quickly reacted to human threat faces by looking away, suggesting that dogs can recognise the expression content and respond as expected as per the dog species-specific repertoire (i.e., averted gaze in dog–dog interactions is widely used in averting visual threat, Bradshaw and Nott 1995). Additionally, this ability to process human facial expressions seems to be influenced by the quality and amount of exposure to human faces in general, and, particularly, if these faces are familiar or unfamiliar (e.g., owner vs stranger, Barber et al. 2016).

Overall, these studies show not only that dogs are attentive to humans (and conspecifics), but also that they are perceiving and reacting to cues of emotion in their social environment. Dogs can also visually discriminate (at least some common) facial expressions of emotion and infer or respond to these emotion cues accordingly, and some of their perception mechanisms seem to be similar to those of humans (e.g., configural process in reading faces and facial expressions). However, there is a lack of comparative studies leaving several important gaps in our knowledge concerning the differences and similarities between how dogs perceive other dogs vs. humans (see Table S1 in the Supplementary Text for examples of studies). Comparative studies of how wolves perceive emotion cues in conspecifics and humans are also needed if we wish to disentangle domestication and ontogenetic effects.

## What methodologies have been used to assess the perception of emotion cues in dogs?

In order to study the perception of emotion cues in dogs, researchers need to conceptually define and then design stimuli that contain these emotion cues (e.g., facial expressions). However, we need to recognise the difficulty, even in humans, of actually measuring emotion responses, due to the highly subjective nature of emotions (see “A brief summary of the nature of emotion and its perceptual processes”). Most studies of human emotion (regardless of whether they assess perception, expression or experience) rely on self-report (i.e., explicit processes) in some form or another, which presents a range of issues (Hofmann et al. 2005; Stone et al. 1999) (see S2 in the Supplementary Text for why implicit measures are better than explicit ones, particularly for dog studies). Nonetheless, technological and scientific advances are opening up possibilities of measuring emotion perception in more detail and using more controlled and systematic stimuli, while achieving better ecological validity. Next, we describe and critique the most common methodologies in dog studies, organised according to common biological indicators of emotion, and whenever necessary for understanding the method used or the critique to the method, we broadly report how these have advanced our understanding of how dogs perceive emotion cues.

### Neurophysiological correlates

By using fMRI in awake unrestrained dogs, researchers have been identifying which brain regions are activated when perceiving a variety of stimuli, including faces, voices, and gestures (Berns et al. 2012; Boch et al. 2021a, 2021b; Cook et al. 2014; Cuaya et al. 2016; Dilks et al. 2015; Karl et al. 2020). Whilst this methodology clarifies perceptual mechanisms at the brain level, it depends on both a substantial volume and prolonged period of activation for the signal to be detected. Small transient responses and regions will not be detected, which might be problematic when looking into low activation perceptual mechanisms of emotion cues. Nonetheless, fMRI studies have successfully shown how the dog brain responds to the emotion content of the human voice (reviewed in Andics and Miklósi 2018) and face (Karl et al. 2020; Thompkins et al. 2018, 2021). This technology has also shown that different regions of the dog cortex process dog vs. human facial expressions and that these regions (Thompkins et al. 2018, 2021), in dogs seem to be analogous to those found in humans, suggesting the existence of shared ancient neural networks for emotion cue perception (Haxby et al. 2000; Thompkins et al. 2021). Whether dogs have a specific brain region for face processing is less clear, with some fMRI studies finding a dog face region (Cuaya et al. 2016; Dilks et al. 2015; Thompkins et al. 2018), while others do not (Bunford et al. 2020; Szabó et al. 2020). Bunford and colleagues (2020) suggested that the inconsistency of results may be due to sensitivity of analysis, contrasts used and/or data analysis. As such, even though dog studies with fMRI have shown replicability (Berns et al. 2013), they are also a technically highly demanding method that still needs fine-tuning at both methodological and conceptual levels (Huber and Lamm 2017; Thompkins et al. 2016). For example, event-related experimental designs in fMRI with few trials per condition (such as in Thompkins et al. 2021) lead to issues of low signal-to-noise ratio and statistical underpower, and hence typically need very large trial numbers (~ 50–100 per condition) to compensate. Whilst these studies give us unique direct insight into the activity of the dog brain when looking at emotion cues, fMRI is perhaps better used in combination with other methods (Karl et al. 2020) or taken cautiously until greater consensus on its value and limitations is achieved.

Another method, fNIRS (functional Near-InfraRed Spectroscopy), that similarly to fMRI was first used in the early 90’s to measure human brain cortex activity (Ferrari and Quaresima 2012), has been used successfully only once in dogs to understand how their brains respond to visual and tactile stimuli (Gygax et al. 2015). In humans, fNIRS has been proposed as a good method for investigating emotion processing (Balconi et al. 2015) and thus, might be a good complementary method to fMRI to investigate emotion cues perception in dogs.

Surprisingly, the first established method to measure human cortical brain activity, the EEG (electroencephalogram, Shipton 1975), has only recently been used to measure dogs’ cortical activity related to emotion cues processing (Kujala et al. 2020). EEG can complement fMRI data since it may be more sensitive to shorter periods of activity. Indeed, Kujala et al. study (2020) showed temporal resolution analogies with humans when dogs processed facial cues of emotion: threatening conspecific faces triggered strong “preconscious” responses with 30–40 ms response latency (typically < 75 ms response latency for visual stimuli in dogs, Törnqvist et al. 2013), while other facial expressions were detected slightly later (127–170 ms) and are closer to “conscious” human responses.

Despite their technical demands in terms of equipment, dog training and data analysis, these are certainly valuable methods for non-invasive studies of dog emotion perception. Particularly in comparative studies with humans, neurophysiological measures and their correlates (e.g., with behaviour) provide important measures of how dogs perceive emotion cues.

### Systemic physiological correlates

The autonomic responses that regulate, for example, endocrine and stress responses (HPA axis, e.g., Mormède et al. 2007) can be measured through a variety of techniques in order to understand how individuals respond internally to particular emotion cues or environmental triggers. Changes in cortisol, oxytocin, heart rate, and temperature are examples of widely used indicators of internal states in dogs, that can potentially be measured and/or manipulated non-invasively (e.g., by using salivary sampling, nasal administration, and external monitors; Barber et al. 2017; Buttner 2016; Katayama et al. 2016; Kis et al. 2015; Kuhne et al. 2014; McGowan et al. 2018; Siniscalchi et al. 2018a, b). Very recently, tear volume has also been examined in dogs as a new physiological indicator (Murata et al. 2022). Since these physiological indicators are correlated with internal states, they allow us to investigate perceptual processes when an individual is exposed to emotion cues. For example, in dogs cortisol increase is correlated with negative arousal (e.g., after an acute stress: Chmelíková et al. 2020) and oxytocin increase is correlated with positive arousal (e.g., after affiliative interactions with humans: MacLean et al. 2017); Hence, with adequate controls in place, these responses can potentially be used to determine if dogs perceive certain emotion cues in a positive or negative way. Conversely, we can also examine how perceptual processes might be modulated by inducing changes in these physiological indicators, such as by administering intranasal oxytocin (Kis et al. 2015).

Oxytocin, with its social bonding role (Romero et al. 2014), has also received particular recent interest due to its function in modulating fundamental emotion processes (e.g., attention to facial expressions), and thus how it might facilitate dogs’ interspecific socio-cognitive abilities (Buttner 2016; Kikusui et al. 2019). The application of oxytocin seems to result in a marked change in gazing pattern to human facial expressions, with elimination of gaze bias towards the eyes in “happy faces” and decreased fixation on “angry faces” (Kis et al. 2017; Somppi et al. 2017). Kis et al. (2017) suggested this oxytocin effect is due to fear reduction, and thus less attention paid to the eyes as a relevant threat cue. Other authors (e.g., Macchitella et al. 2017) suggested a more general mechanism involving the creation of a positive expectation bias towards human behaviour to facilitate the interpretation of the observed cues.

Studies measuring heart rate in dogs also show significant effects when dogs are exposed to human emotion cues. For example, heart rate increased and heart rate variability decreased when dogs were exposed to a threatening stranger (i.e., fixed gaze on the dog while approaching, Gácsi et al. 2013). Similarly, in another study (Barber et al. 2017), dogs gazing at human “angry faces” showed the highest increase in heart rate when compared to neutral, followed by “happy faces”. On the other hand, “sad faces” decreased heart rate in comparison to “neutral faces”. Since both “happy” and “angry faces” triggered an increase in heart rate and “sad faces” led to a decrease, it suggests heart rate is a better correlate of arousal or emotion intensity, which when used with behavioural indicators of emotion quality might be useful to disentangle these potentially confounding factors.

Infrared thermography (IRT) has also successful been used to record surface temperature changes in different parts of the body (e.g., eye, ears) when dogs were subjected to positive and negative situations (e.g., veterinarian examination, Travain et al. 2015, Csoltova et al. 2017; owner separation, Riemer et al. 2016; receiving preferred food, Travain et al. 2016). In the negative situations, eye temperature tended to increase, whilst ear temperature decreased (Riemer et al. 2016). However, in another study (Fukuzawa et al. 2016) in which strangers or owners approached dogs with neutral or smiling facial expressions, no differences were found between conditions. In this latter study, IRT was only used 2 min after the approach action, so perhaps thermal changes are detectable only whilst a particular positive or negative stimulus is present.

Despite its value as a direct link to the internal changes during emotions in individuals, physiological correlates on their own are extremely difficult to interpret due to both individual variation and numerous co-variates (e.g., time of day, age of the dog, etc.), which demand intense protocol standardisation (Chmelíková et al. 2020). Furthermore, they tend to vary in response to multiple stimuli that may be unrelated to emotions (e.g., physical or cognitive activity level: Colussi et al. 2018), and often produce conflicting results (e.g., MacLean et al. 2017 vs. Powell et al. 2019). Physiological correlates, while potentially useful to assess how individuals perceive emotion cues in others, require much more research and should only be used in conjunction with other measures, in particular behavioural indicators.

### Cognitive and behavioural measures

**Fig. 4:**
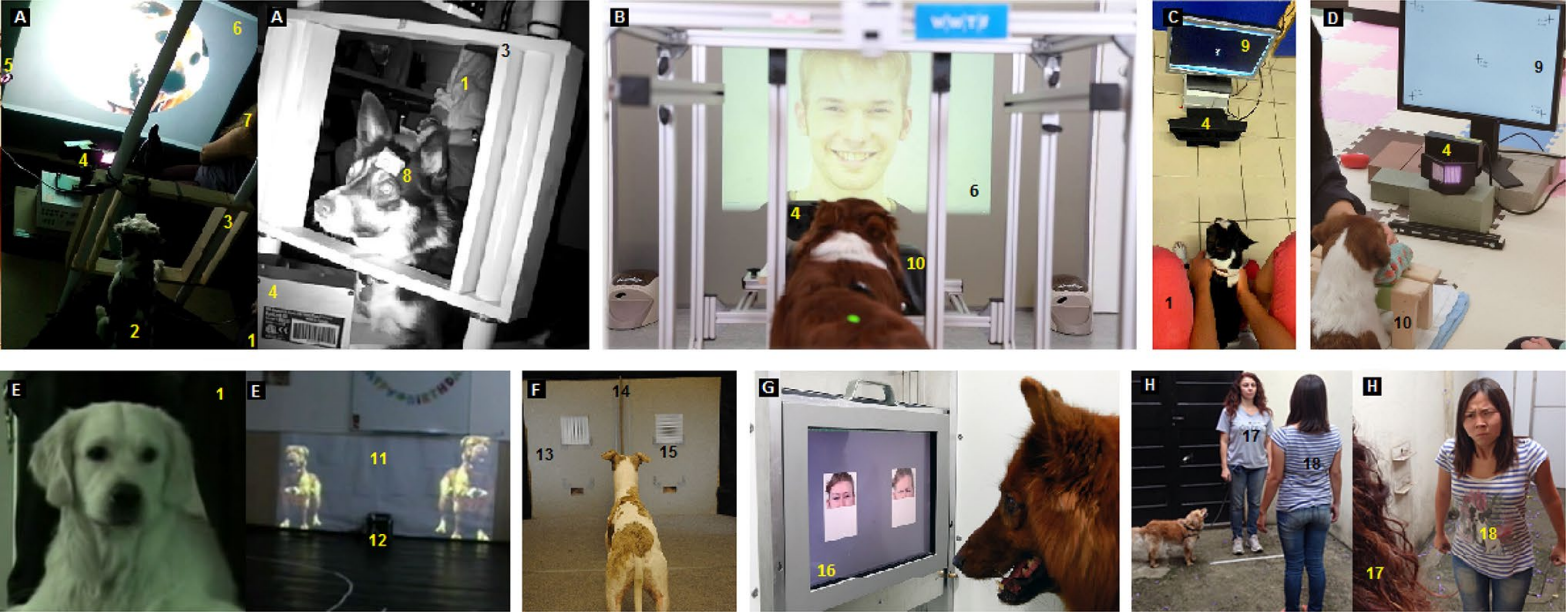
Examples of various experimental setups and equipment that can be used to investigate perception of emotion cues in dogs (pictures selected may not be from studies on perception of emotion cues as they are for illustrative purposes only). Experimental setups from: **A** Correia-Caeiro et al. (2020, 2021), **B** Barber et al. (2016), **C** Kis et al. (2017), **D** Ogura et al. (2020), **E** Faragó et al. (2010), **F** Lind et al. (2017), **G** Muller et al. (2015), **H** Albuquerque et al. (2021). Image 4-B and 4-G courtesy of Ludwig Huber. **1**: Owner sitting behind or next to the dog, **2**: Dog participant, **3**: Frame for free-range of motion for the eye-tracker, **4**: Eye-tracker camera, **5**: Infrared camera, **6**: Back-projected stimuli, **7**: Experimenter facing away from the dog, **8**: Eye-tracker target for eye triangulation, **9**: LCD display, **10**: Chin-rest, **11**: Canvas with front-projected stimuli, **12**: Speaker, **13**: Grey board to pin stimuli, **14**: Separator between stimuli pair, **15**: Paper printed stimuli, **16**: Touchscreen, **17**: Owner involved in the task, **18**: Experimenter performing emotional displays for the task

These are probably the most common indicators used for measuring canine perception of emotion cues, due to relative ease of implementation in terms of methodology and generally lower ethical concerns. By using a wide variety of experimental setups and equipment (Fig. 4, Table 1), researchers can systematically record how individuals respond to a controlled stimulus, and thus inferences can be made about their perceptual abilities.

**Table 1:** Comparison of experimental setups and equipment that can be used to investigate perception of emotion cues in dogs (see Fig. 4 for pictures of each experimental setup)

Figure 4 cross-reference	Study reference	Experimental paradigm/data recording	Stimuli presentation method	Pre-experiment training?	Owner present and blinded?	Experimenter present?
A	Correia-Caeiro et al. (2020, 2021)	Eyelink 1000 Plus (SR Research) eye-tracker on free moving head mode	Back-projection screen	No	Yes	Yes, facing away from the dog and stimulus
B	Barber et al. (2016)	Eyelink 1000 eye-tracker on chin rest mode	Back-projection screen	Trained dogs to place head still on the chin rest	Owner sitting behind the dog	No
C	Kis et al. (2017)	Tobii X50 eye-tracker on free moving head mode	17-inch LCD screen	No	Owner sitting behind the dog, holding the dog’s body	No
D	Ogura et al. (2020)	ISCAN ETL-300-HD eye-tracker on chin rest mode	21.3-inch LCD screen	No	Owner outside the room	Two experimenters present instructing and/or holding the dog's head to rest on the chin-rest
E	Faragó et al. (2010)	Intermodal Visual Paired Comparison (IVPC), looking time recorded on camera and manually scored	Front projection canvas	No	Owner behind the dog wearing headphones	No
F	Lind et al. (2017)	Two-choice discrimination paradigm	Printed on paper and pinned to a board	Pre-training of dogs to associate one stimulus with reward	Owner behind the dog looking down	No
G	Muller et al. (2015)	Two-choice discrimination paradigm on a touchscreen	Touchscreen	Pre-training of dogs to use the touchscreen and to associate one stimulus with reward	Owner present out of view from the dog	Yes
H	Partial experimental setup from Albuquerque et al. (2021)	Effect of human social cues on a “V” detour task, behaviour recorded on camera and manually scored	Human experimenter to perform emotional displays and demonstrate how to solve a task	No	Owner present and instructed to play a role in the task	Two experimenters present

For example, in the lateralisation studies such as those mentioned in “What is known about how dogs visually perceive emotion cues?” (Siniscalchi et al. 2010, 2013), dogs produced particular behaviours with a side bias towards valenced stimuli (e.g., head turn left when seeing human facial expressions). This relationship between brain hemisphere bias in valence processing and lateralised behaviour might give us insight into how the dog might be perceiving a certain stimulus, including stimuli featuring emotion cues. However, exceptions and/or inconsistencies in the side bias studies (e.g., head turn left for negative but also “happy faces”) between behavioural and neural correlates remain to be elucidated before its further use for the assessment of perception of emotion cues.

Another widely used behavioural measure in dog cognition studies is gaze or body orientation towards a stimulus; Despite these measures not always recording exclusively active observation or attention (but may also record blank stares (Aslin 2007) or gaze avoidance), how individuals observe their environment often provides important information on perception and processing of emotion cues. Gaze behaviour can be recorded and interpreted through cognitive paradigms and/or eye-trackers (Fig. 4, Table 1).

Following on from infant studies, classical or variations of cognitive paradigms such as Intermodal Visual Paired Comparison (IVPC, Albuquerque et al. 2016, Fig. 4-E) and Expectancy Violation (EV, Adachi et al. 2007) have been used in studies with dogs to assess different aspects of facial processing. IVPC has been used to test if dogs can extract and integrate emotion cues from different modalities (e.g., voice and facial expression), and thus recognise the associated emotion (Albuquerque et al. 2016). These studies typically compare the duration of the natural gaze of dogs towards each of two visual stimuli (e.g., facial expressions pictures) presented side-by-side following an auditory stimulus (e.g., voice), to infer how individuals process these stimuli (Fig. 4-E). Similarly, EV has been used in dogs to test cross-modal recognition of owner identification (Adachi et al. 2007) and other dogs as a species (Mongillo et al. 2021), but not yet for emotion cues (but see (Nakamura et al. 2018) for EV used with horses for successful emotion cues recognition). EV studies repeatedly present one stimulus followed by a second stimulus (e.g., congruent or incongruent image) and then compare looking times between conditions. Both experimental paradigms test internal representations of concepts, but are based on slightly different processes: IVPC is based on the integration of cues from two modalities (found when individuals face two simultaneous visual stimuli and prefer to look at matching audio-visual stimuli), while in EV individuals are assumed to integrate the audio-visual cues and look more at the non-matching stimuli due to being presented with cues that cannot exist together (found when individuals look more at incongruent stimuli). Whilst these methods can be easily implemented to investigate dog perception of emotion cues, these may also be a limited method which traditionally has relied on manually coding eye movements in dogs, (a task notoriously difficult due to the iris usually being dark colour and without a visible white sclera). A better approach from a methodological point of view (but perhaps more expensive and harder to implement), is the combination of eye-tracking as a recording method and IVPC and EV as experimental paradigms, but no study has yet used them in combination. It is also difficult to objectively interpret what the preferential looking actually means, which can be both interpreted as visual preference for congruency, because it integrates matching information (e.g., voice and face of owner), or preference for incongruency, because it is unexpected and hence draws more attention (Winters et al. 2015).

Within the studies looking at perception of facial expressions, two pieces of equipment have perhaps proved more informative regarding dogs’ perceptual worlds: touchscreens (Fig. 4-G) and eye-trackers (Fig. 4-A-D). Touchscreens have been widely used for examining many cognitive and perceptual abilities in dogs (e.g., categorisation, Range et al. 2008; face processing, Pitteri et al. 2014b; learning, Wallis et al. 2016; illusion perception, Keep et al. 2018), but rarely for emotion cue perception (Müller et al. 2015). This latter study showed that dogs are able to discriminate human facial expressions. However, perhaps due to restrictions in sample size (i.e., not all dogs can easily learn the task) or in the time needed for training, despite their huge potential for cognition and emotion perception studies, touchscreens are not yet used extensively in this area. By contrast, eye-trackers have been used for dogs in an increasing number of studies (Gergely et al. 2019; Karl et al. 2019; Ogura et al. 2020; Park et al. 2019; Rossi et al. 2014; Somppi et al. 2012, 2014; Téglás et al. 2012; Törnqvist et al. 2015, 2020; Völter et al. 2020) and specifically to investigate emotion cue perception (Barber et al. 2016; Correia-Caeiro et al. 2020, 2021; Karl et al. 2020; Kis et al. 2017; Somppi et al. 2016, 2017). These studies have investigated not only how dogs read facial expressions (and in one study also body expressions, Correia-Caeiro et al. 2021), but also what factors modulate this behaviour (e.g., experience with humans: Barber et al. 2016) and how this influences the human–dog relationship (e.g., Karl et al. 2020). The advent of mobile eye-tracking technology (Pelgrim et al. 2022; Williams et al. 2011) can extend this work to more ecologically valid settings with real rather than recorded stimuli. While most modern eye-trackers (i.e., based on detecting near-infrared pupil and cornea reflections) have been specifically developed for the human eye, its use with dogs has been remarkably successful, probably due to the similarity between the human and dog pupil and cornea-generated reflections (Barber et al. 2020; Somppi et al. 2012). However, dogs do present some differences in their visual system, such as a horizontally wider fovea (Beltran et al. 2014) and different eye movements (Park et al. 2019), but it is still unclear if or how these differences may impact visual perception of emotion cues.

## What are the limitations and challenges to investigate the perception of emotion cues in dogs?

When compared to neurophysiological and systemic physiological correlates, behavioural and cognitive correlates are perhaps the most prone to issues of subjectivity and observer biases, and thus the choice of observational tool and use of controls become crucial to the evaluation of experimental validity. Fortunately, there has been a rapid technological and scientific progress of methodologies such as eye-tracking to investigate dog perception of emotion cues, accompanied by many practical advantages (e.g., ethical, ease of use). Nonetheless, other issues still need some further discussion to allow successful replication of studies, such as the use of consistent and precise definitions or what variables are being measured (such as quantification of facial movement or anatomically-driven Areas of Interest—AOIs in eye-tracking data analysis). Instruments such as DogFACS (Waller et al. 2013) allow both standardisation of facial cues of emotion when designing/selecting experimental stimuli and objective measurement of facial responses to emotive stimuli. Likewise, eye-trackers (e.g., Somppi et al. 2016) precisely collect an extensive array of metrics related to eye movements and pupil size (Völter and Huber 2021, 2022) that can be objectively represented relative to the stimulus being viewed (e.g., as fixation points and heat maps, Holmqvist et al. 2011; Kowler 2011). However, it is important to appreciate methodological constraints that may be present, not only when using certain equipment, experimental paradigms or when measuring certain indicators, but also when using dogs as a model species. Therefore, in this section, we critically consider some of the most pervasive issues in the dog perception/cognition literature and suggest some best practices and recommendations following from each issue. We also suggest examples of research questions that are needed to address issues arising from the methodologies used and its challenges (summarised in Table 2). In this section, we also discuss some of the limitations and challenges further, to assist researchers reviewing previous work or planning future studies with dogs, especially in relation to dog emotion cue perception.

**Table 2:** Points to consider and recommendations to design experimental stimuli and protocols, and tailor it to each particular dog as necessary (middle columns). Suggestions of research questions for future studies that may answer particular methodological 592 challenges are also listed (column on the right). Points to consider and suggestions for future studies (both novel questions or 593 deepening of published questions) are organised by the features more prone to challenges (column on the left)

Features prone to challenges	Points to consider	Recommendations	Examples of future research questions
6.1. Breed, individual differences, and the umwelt of each dog	Diversity of dogs as a species	Sample larger diversity of dog types regarding breed, age, sex, cephalic type, facial morphology, human environment, life history, etc.	How does perception of emotion cues develop and vary over the lifetime of the individual?
	Individual differences within dog types	Consider the umwelt of the dog that may vary within dog types regarding temperament, personality, motivation, mood, etc. Assess preferred rewards (e.g., food vs praise vs play) to ensure optimal motivation and attention during task Use validated psychometric scales and/or behavioural tests to assess individual differences Assess sensitivity to rewards and aversives with e.g., PANAS, Positive and Negative Activation Scale (Sheppard and Mills 2002) to assess each dog’s emotional predispositions and avoid sampling bias or excluding dogs Assess temperament and impulsivity to understand to which dogs the task provides an inherent reward (e.g., play with a human), and which dogs require external rewards (e.g., treat) to increase extrinsic motivation (Deci et al. 1999) Keep motivation and focus high, whilst keeping over habituation and boredom at a minimum (e.g., allowing the dog to leave/stop the experiment at any time for a short break, keep trials/sessions short and stimuli as varied as possible)	How does perception of emotion cues vary between different temperaments?
	Differences between dogs and humans	Consider differences between dogs and humans when adapting experiments developed for humans and regarding sensorial abilities (visual and other) Control for other sensorial contaminants and influxes in the testing environment, which may go undetected by humans but bias dog behaviour (e.g., odour, magnetism, temperature)	To what extent do dogs and humans use similar mechanisms for processing emotion cues of both conspecifics and heterospecifics? Investigate sensory abilities present in dogs but not in humans: Does magnetism or temperature affect dog's visual perceptual mechanisms?
6.2. Experimental design: controlling variables whilst maintaining ecological validity	Presence of the owner	Allow presence of the owner, since 1) this makes the controlled environment of a laboratory more naturalistic and also emotionally equable for different subjects; 2) owners can act as secure bases for dogs in novel environments and when encountering strangers such as the experimenter (Gácsi et al. 2013) But also blind owners (both metaphorically—withhold experiment goal until its end, and literally—use blindfold, earplugs), as owner’s inadvertent cuing must be controlled to avoid Clever Hans effects (Miklösi et al. 1998; Schmidjell et al. 2012)	How does the presence of the owner affect the performance of dogs in tasks of visual perception of emotion cues?
	Collection of dog spontaneous responses and ecological validity	Allow for free full body movement responses to the stimulus (e.g., tail wagging, head turns), since the absence of natural responses may impact perceptual processes Give preference to naturalistic experimental protocol steps (luring/holding lightly vs. extensive training for immobilisation), and avoid conditioned or emotionally primed responses If less naturalistic steps are absolutely needed (e.g., immobilisation in fMRI or to assess eye saccades), discuss how these may have impacted the results (e.g., not moving the head when perceiving emotional cues, which are known to cause head turns in dogs (Siniscalchi et al. 2010, 2013)) But also control for increased random error and risk of correlated systematic error, which can make the correct and precise identification of the influential variable(s) more difficult. Random error effects may mask important effects that would be significant in a more controlled environment, and systematic error associated with other factors may lead to inaccurate associations Discuss how effects found in a highly controlled setting would stand in a real-life scenario. More controlled experiments (e.g., in the lab) facilitate equipment handling and ensure important but small effects are not masked by other variables, but may lose ecological validity Consider the thermal and magnetic properties of the equipment used (e.g., visual display units, fMRI, Fig. 5) with dogs as a potential confound in experiments (since dogs can sense these)	Does immobilisation of the dog affect the perception of emotion cues? How does the wide range of experimental and stimuli properties (Fig. 4, Table 1) influence perception of emotion cues in dogs? For example, real-life demonstrators vs. video, spontaneous vs. posed emotion cues, passive viewing vs. task engaged, trained for remaining immobile vs. allowing movement (e.g., tail or head turns) Does the equipment used in experimental setups with thermal and magnetic emissions impact the stimuli perception or the dog performance?
6.3. Experimental stimuli: spontaneity and validity of stimuli, but with well-defined categories and objectively measured cues	Selection and design of stimuli	Thoroughly define and justify the conceptual basis for the stimuli selection and design (e.g., psychobiological approach, context and triggers used to induce facial expression, quantification of cues that match the dog behavioural repertoire) Avoid face-centric stimuli and include body postures and gestures, which are particularly important for dogs, according to their natural behaviour and recent eye-tracking evidence (Correia-Caeiro et al. 2021)—this will counter the evident face publication bias and consider the umwelt of dogs Avoid the use of the exact same triggers to create stimuli featuring humans and dogs; Give preference to functional equivalent triggers (e.g., adult humans are generally not afraid of thunderstorms (Silverman et al. 2001), while dogs often are, at a clinical level (Lopes Fagundes et al. 2018; McPeake et al. 2017; Overall et al. 2001), hence thunderstorms may be a good trigger for dog fearful behaviour, but not for humans) Avoid (or be particularly cautious with) the use of emotion categories and corresponding behaviours/visual cues common in humans but that may not be found in dogs (or at least not in the same form), including emotion categories and respective cues that are currently still being debated in canine science (e.g., guilt (Ostojić et al. 2015), jealousy (Cook et al. 2018; Karl et al. 2021)) And vice-versa, use emotion categories that are more common/relevant in dogs and have associated emotion cues in dogs but not humans. For example, positive anticipation cues are present in dogs but not humans	What are the differences in potential emotional states and its associated cues between dogs and humans? What contexts and triggers are ideal to collect stimuli for perception of emotion cues in dogs? What potential emotional states may be triggered by sensing magnetism or a distant source of heat and are there any cues displayed during these states?
	Classification and description of stimuli	Avoid emotion labels for stimuli, since these are subjective and too broad Avoid using anthropomorphic/anthropocentric emotion categories, i.e., based solely on human research (e.g., from facial expressions), particularly if there haven’t been fundamental studies demonstrating these to be associated with a particular type of display in dogs Explicitly describe the context in which the stimuli were collected (i.e., dog growling during food competition/territory defence, etc.) Quantify the emotion cues observed in the stimuli (e.g., how many/which/duration of AUs/gestures/postures), by for example using tools such as DogFACS (Waller et al. 2013) or DogBAPS (Huber et al. 2018)	Exactly what AUs/gestures/postures (e.g., facial and bodily expressions) dogs display in response to different emotional triggers? (also very little studied in dogs)
	Validation of stimuli	Give preference to ecological (i.e., pertaining to appropriate environment for the species) and evolutionary (i.e., pertaining to survival value for the species) validity, whilst considering the species natural behaviour, ecology and motivation (Tomasello and Call 2008), in order to ensure laboratory findings can be generalised to the outside world Avoid asking vague “expert opinion” (or a sample of random human observers) to validate the stimuli regarding the emotion as the only validation step—this will likely just incur in circular reasoning and confirm human observers biases Instead validate the stimuli by asking experts to independently quantify cues present in the stimuli and/or describe the context in which the cues were produced (see previous point about classification and description of stimuli)—this will assess agreement on objective and measurable cues instead of subjective impressions Describe in detail in the methods section how the experts validated the stimuli as appropriate for the tested effect (e.g., a “happy dog face” needs to display “relaxed open mouths”, “lip corners retracted”, and absence of “ears backwards”)	
	Presentation of stimuli	Present stimuli in an ecological valid way (e.g., facial expressions not at the dog’s eye level) Give preference to video as stimuli (Correia-Caeiro et al. 2021; Karl et al. 2020) and avoid static pictures, since the former includes onset, apex and offset of a visual cue (e.g., facial expression), natural timing, symmetry, intensity, etc. that the dog is more familiar with in their daily life Give preference to spontaneous stimuli and avoid posed stimuli (e.g., real-life demonstrators will vary in their behaviour and will have posed behaviours, spontaneous facial expressions differ from posed facial expressions) But also match the specs of the equipment used for stimuli presentation to the visual abilities/needs of dogs (e.g., screen high refresh rate)	Is there an impact when stimuli are placed at the dog’s eye level when in real-life it is not (e.g., over-inflation of face which may modify eye movements)? Does dynamic information change perception of emotion cues in dogs as it does for humans? How do canine displays performed on command differ from spontaneous displays?

**Fig. 5:**
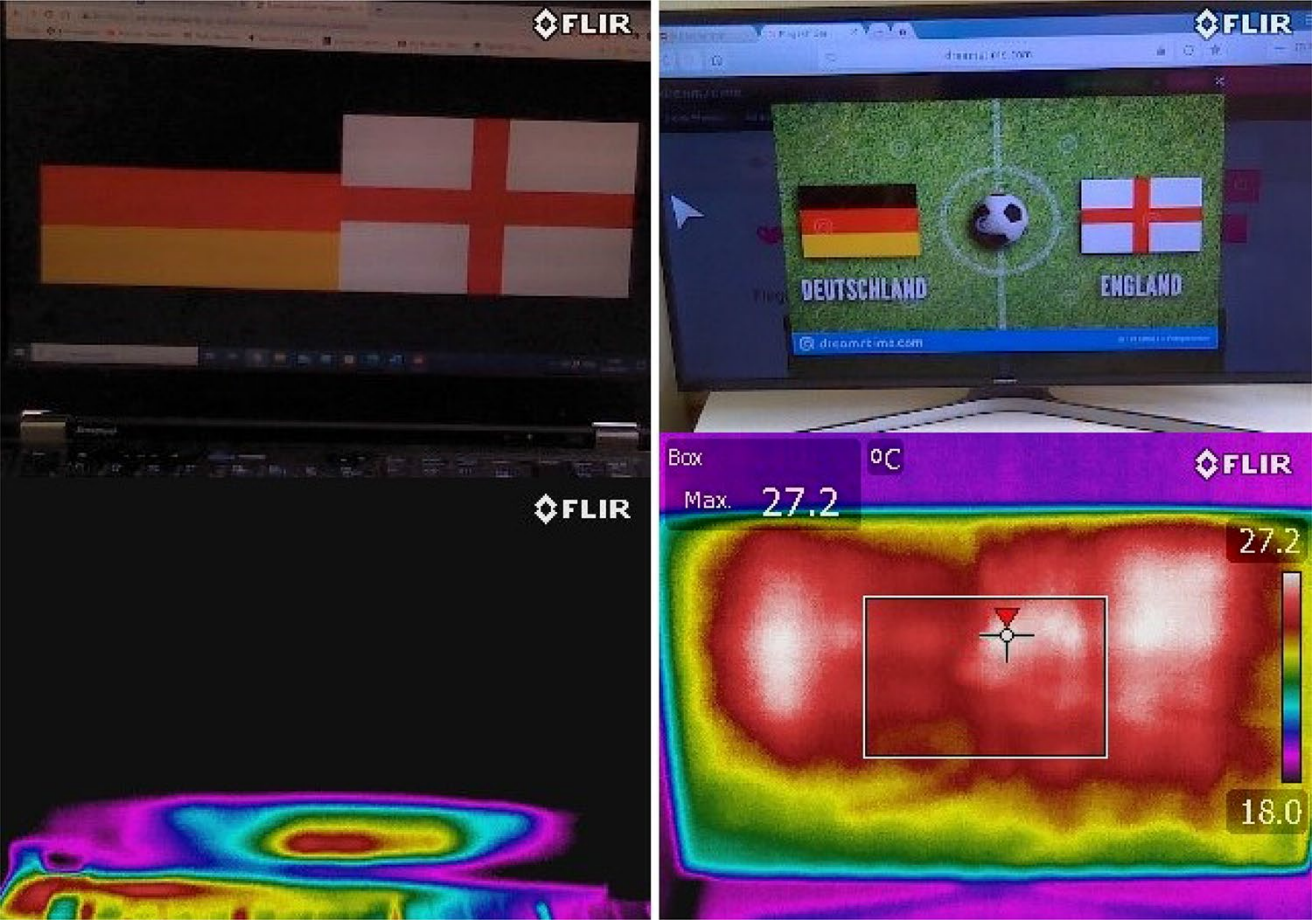
Top left: Laptop screen displaying a coloured image in the visible spectrum; Bottom left: thermal image of same laptop screen after 5 min—the thermal differential is associated with the keyboard and screen base; Top right: LED monitor displaying a coloured image in the visible spectrum; Bottom right: thermal image of same LED monitor after 5 min, highlighting thermal gradient associated with different colours. Image courtesy of Tim Simon

### Breed and individual differences

Dogs are often viewed as a homogenous species, despite their wide morphological, genetic, and behavioural variation, which makes the generalisation of results to “dogs” questionable in many circumstances, since the sample is not representative of all types of dog. Added to this are potential lifespan changes that might not be apparent if the sample is not truly representative of all types of dog of all ages. Differences in how dogs perceive their (social and non-social) environment have been found with regards to a dog’s skull length, breed, sex and/or age (Bognár et al. 2018; Correia-Caeiro et al. 2021; Heberlein et al. 2017; Jakovcevic et al. 2010; Scandurra et al. 2018). For example, hunting dogs were more attentive to their owners than shepherd dogs (Heberlein et al. 2017), while aging led to decreased attention to human facial expressions (Correia-Caeiro et al. 2021).

Functional anatomical differences in sensory abilities between types of dog (e.g., see Barber et al. 2020 for a comprehensive review of the visual system of dogs relative to humans and its implications) mean that the social environment might be perceived very differently, regardless of any central capacities; i.e., the brain may be receiving very different stimuli which may affect the subsequent processing and behavioural responses. In addition, dogs live in a variety of human environments (companion/urban vs free-ranging/village dogs), which seems to impact, for example, sociability (Bhattacharjee et al. 2021) but not some perceptual abilities (Bhattacharjee et al. 2020), although companion dogs have been much more studied than free-ranging dogs.

In addition, factors related to an individual’s life history, experience, personality, etc. may also shape the way individuals perceive their social environment, particularly emotion cues. These raise the importance of the concept of *umwelt* (Uexküll 1957) in animal communication (Manning et al. 2004; Partan and Marler 2002; Uexküll 1957; Uexküll and Mackinnon 1926), and likewise in dog emotion perception, in which the subjective phenomenal world varies between individuals. The umwelt of an individual might influence, for example, sensitivity to certain cues or the motivation and attention needed to perform a task. This inevitably generates a sample bias by selecting dogs who complete the task. Differences in temperament (and also impulsivity, Fadel et al. 2016; Wright et al. 2011) may lead to some dogs being more easily trained or intrinsically motivated within the experimental setup (Brady et al. 2018; Cavalli et al. 2018).

While the standardisation of the laboratory environment is often projected as a way of controlling for extraneous variables, in the context of emotion cues perception, it needs to be recognised that two dogs may perceive the sterile laboratory or same experimenter in very different ways (due to their umwelt). One may adapt and the other may perceive it as stressful; accordingly they may be emotionally primed in very different ways and this may affect their attention focus and perception (Burman et al. 2011; Sümegi et al. 2014).

### Experimental design

The balance between controlling variables and maintaining ecological validity is a delicate and challenging one, which must be carefully considered. The problem of highly controlled and “aseptic” laboratory studies is that they might find effects that are of little relevance in the “real world” where many more variables are interacting with the experimental variables of interest. This can lead to problems of replicability, which are a concern not only in this area, but in the wider area of psychology (Farrar et al. 2021; Open Science Collaboration 2015; Tecwyn 2021). Furthermore, it also means research is focused on what we can measure in the laboratory rather than what might be ecologically important.

Typically, emotion cue perception experiments with dogs tend to feature a passive visualisation of many trials and repetitions of relatively similar stimulus (e.g., facial expressions), which might lead to habituation and/or boredom, and subsequently affect attentional mechanisms which are crucial for such perceptual experiments. There are also protocol differences in how dogs are expected to participate in the experiment (Fig. 4, Table 1). For instance, in some eye-tracking studies the dogs are lightly physically held in place (e.g., Fig. 4-C, Kis et al. 2017) or lured to lie still (e.g., Fig. 4-A, Correia-Caeiro et al. 2020) on the day of the experiment, but in others, dogs are trained for several weeks/months throughout several stages before the experimental stage in order to remain immobile and place their heads on a chin rest to face the screen (Karl et al. 2019; Somppi et al. 2012). While an eye-tracker protocol with training is preferred due to limitations in certain eye-tracker models that do not allow head movements or whenever high-accuracy of eye movements is needed, protocol without training has a range of advantages, including less time/work invested before the testing stage, fewer exclusions of individuals that might not reach criteria during training (and thus better representation of the species), as well as allowing for unconditioned responses and more naturalistic behaviour (allowing head turns for aversive stimulus, tail wagging, etc.). In particular, free head movements might be important when measuring eye movements as head fixation may impact perceptual and cognitive processes. For example, eye movements differ in humans (Collewijn et al. 1992) and mice (Meyer et al. 2020) between head fixed and head free setups. A non-training protocol that allow individuals to choose whether to watch the stimuli or not may also incur in lower data/calibration quality or data loss and the need to repeat calibration and trials more often, so due consideration to these aspects must be given. Another difference between training and non-training protocols is the time dogs spend watching a stimulus. Whilst with training protocols, individuals are more likely to watch the stimulus (because they were trained to do so and due to the immobilised posture facing the screen) and thus more data points are collected, these may not represent how dogs observe stimuli in real life (e.g., dogs avoid staring at faces of other dogs as this is a threatening signal). On the other hand, non-training protocols allow the individuals to watch or to avoid the stimuli according to natural behaviour, but data collected may be less or with lower quality. A final consideration is that in both cases the preconditioning with rewards might itself create an emotional bias.

The same consideration needs to be given to differences in the degree of active involvement by dogs in the protocol of choice: some simply require passive viewing of stimuli (e.g., eye-tracking, IVPC/EV paradigms), while others request dogs to perform in more complex scenarios in which they need to make choices (e.g., through target approach: Fig. 4-F, touchscreen activation: Fig. 4-G) or take part effectively in social interactions with live demonstrators (e.g., Albuquerque et al. 2021; Buttelmann and Tomasello 2013; Vas et al. 2005). While it can be argued that all scenarios are to some extent naturalistic, since dogs not only passively view emotion cues in social partners but also process these cues when interacting with their social partners, there might be a difference in the cognitive processes recruited when additional cognitive and physical processes are accompanying emotion cue perception.

Another common protocol approach when presenting visual stimuli to dogs is to place all stimuli vertically centred at dog's eye/head level, in order to enhance the chances of detection of stimuli (particularly important in immobile setups, where the head should be at a comfortable angle for the dog for a period of time). However, specifically when presenting human facial expressions, this might be problematic, as dogs do not usually see human faces at eye level in real-life interactions, but instead need to look up on the vertical axis to detect facial cues of emotion (Correia-Caeiro et al. 2021).

Dogs may also make use of senses that humans or other primates are not known to be able to use. For example, recently it was discovered that dogs can sense heat with their noses from a distant source (Bálint et al. 2020) and can sense magnetism both from the Earth’s field and from magnetic objects (Adámková et al. 2017, 2021; Hart et al. 2013; Martini et al. 2018). Visual display units commonly used in experimental setups with dogs vary the temperature distribution across the colour spectrum on the screen (Fig. 5). Heavily magnetic equipment (e.g., fMRI) may also interfere with dog’s perceptual processes. However, very little research has of yet been done on these sensorial modalities in dogs (also see Table 2).

### Experimental stimuli

Whereas humans can produce posed facial expressions (even though these vary in timing, intensity, and complexity in comparison to spontaneous ones, Cohn and Schmidt 2003; Raheja and Gupta 2010), there is no evidence that dogs can “act out” emotion reactions. Even when trained to perform a certain display (Déaux et al. 2015), it is unknown if this represents a faithful reproduction of the spontaneous reaction that would be displayed in a naturalistic context. This poses a problem since, typically, studies of dog (and human) perception use pictures/videos of dogs often displaying stereotypically aggressive/happy facial expressions taken out of context and without any control for the emitted cues. Thus, the experimental stimuli may lack empirical evidence to categorically state that they represent a happy/sad/angry dog. In general, humans are quite poor at classifying dog facial expressions and body postures (Kujala et al. 2012; Meints et al. 2010; Meints 2017; Meints and de Keuster 2009) and this might extend to their selection of appropriate stimuli for emotion cue perception studies. “Expert” agreement on its own, potentially creates a circular reasoning centred on the human perception of what a “happy dog” looks like. The tautology goes like this: there is a general idea that a happy dog looks in a specific way based on broad and non-standardised descriptions of dog behaviour (e.g., Darwin 1896; McGreevy et al. 2012), “experts” agree with each other what is the best example of this particular look, generally without specifying why, and then this is shown to other humans (e.g., participants in a survey) that unsurprisingly, agree with the experts. However, this is basically assuming that the human perception of emotion cues in dogs is interchangeable with the actual emotion experience and thus expression of cues in the dog, which may not be the case. In Bloom and Friedman (2013), the authors created stimuli featuring facial cues of emotion in a single dog to parallel a database of human facial expressions for basic emotions (Ekman 1992; Ekman and Friesen 1976). The dog facial expressions included emotion cues for responses such as disgust, whose neurological, physiological and behavioural correlates have not been studied in dogs. Since the human facial expressions for the basic human emotions have not all been found in dogs, this approach does not have a scientific basis. The opposite may also be true, where some emotions may not have a defined human facial expression. For example, positive anticipation (i.e., reward anticipation) in dogs has strong neurocognitive evidence (Berns et al. 2012; Cook et al. 2016) and is associated with specific facial and ear movements (Bremhorst et al. 2019; Correia-Caeiro et al. 2017). However, in humans, it does not present a stereotypical facial expression, being identified instead by the absence of corrugation movement (Korb et al. 2020).

This attribution of human features to animals (at least without scientific evidence) or if selecting human features as the only ones important to consider when looking at human–dog interactions results in anthropomorphic and/or anthropocentric stimuli. In addition, stimuli that are “stereotypically” human, might also be socially and experientially constructed to some extent (i.e., they are learned and vary across cultures, Barrett 2006; Elfenbein et al. 2007; Jack et al. 2012a, b; Keltner and Haidt 1999). A more naturalistic approach based on investigating what kind of displays are produced when the dog is faced with a potential emotion triggering context along with other evidence to triangulate the emotion may offer a more logical, systematic, and scientific solution (Mills 2017). To gain a deeper understanding of what a “happy” dog truly looks like, recent studies (Bremhorst et al. 2019, 2021; Correia-Caeiro et al. 2017, 2020, 2021; Park and Kim 2020) have applied DogFACS (Waller et al. 2013). This anatomically-based, standardised and objective method of facial coding allows not only validation and precise control of stimuli displayed in perceptual experiments, but also empirical measurements of emotionally-linked facial movements.

Not only is it important to define and capture spontaneous experimental stimuli, but we need to also consider its dynamic nature. Typically, studies of perception of emotion cues in dogs have used static facial expressions “frozen” at a high intensity as stimuli, that suddenly appear on a screen (e.g., Fig. 6 from Barber et al. 2016; Somppi et al. 2016). While high intensity static stimuli might produce a larger response and thus less noisy data due to their visual saliency, such stimuli pose some issues regarding ecological validity. For example, for human facial expressions, high intensity static stimulus is dissimilar from facial expressions displayed in real-life, which have an onset, apex and offset (i.e., appearing/disappearing gradually with very specific timings, or displayed at different, usually much lower intensities, Cohn and Schmidt 2003; Ekman et al. 2002a, b), and omit the dynamic information that is an integral part of facial expression processing (Kilts et al. 2003; Rymarczyk et al. 2016). Hence, when these stimuli are presented to dogs, they may be seen as novel/unusual stimuli or harder to be processed due to lack of experience with such stimuli. While both video and static image stimuli may have limitations regarding visual properties (2D, colour use, refresh rate, etc.), these type of stimuli are easier to control and some of their visual properties can be adapted (e.g., using higher refresh rate), However, in some studies on how dogs perceive emotion cues, real-life human demonstrators have been used to display the stimuli (e.g., facial and vocal expressions in the social referencing paradigm (Merola et al. 2012, 2013, 2014) or its effect on learning tasks (Albuquerque et al. 2021)). Whilst real-life demonstrators might better engage and motivate dogs in the experimental tasks, it also introduces varying degrees of lack of control and thus validity, such as in the difficulty involved in fully blinding demonstrators, in the display of posed cues, in the ability to repeat identical cues between trials, or in what cues exactly dogs are taking from the demonstrators.

**Fig. 6:**
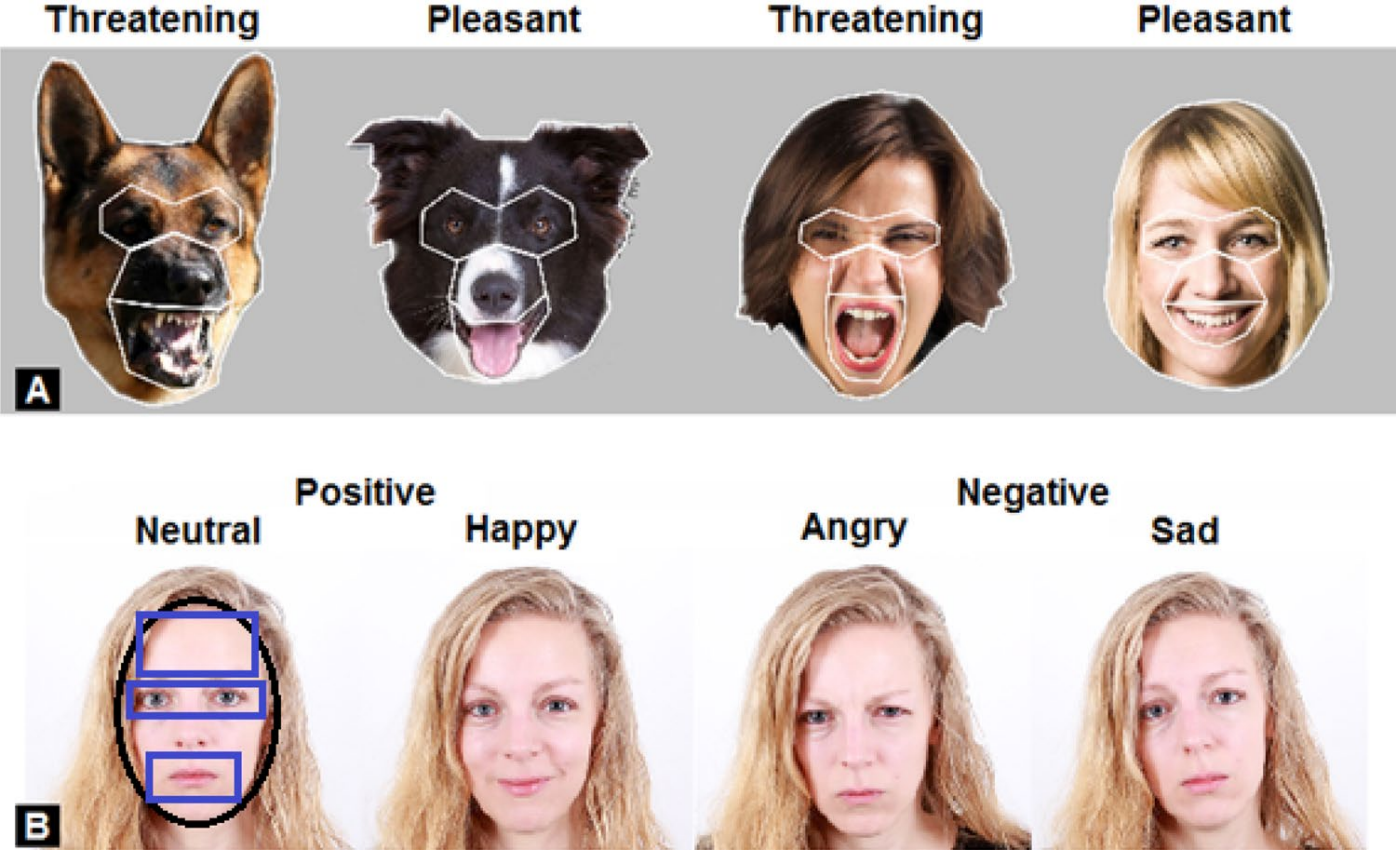
Examples of stimuli used in experiments aimed at investigating dog perception of facial expressions, with emotion labels and AOIs selected by the respective authors. A—Areas of Interest—AOIs labelled as “eyes”, “midface”, “mouth”, and “whole face”, adapted from Somppi et al. (2016), B—AOIs labelled as “forehead”, “eyes”, “mouth”, and “face rest”, adapted from Barber et al. (2016)

Moreover, research has concentrated on how dogs perceive facial emotion cues, due to the face being crucial in human–human interactions, but little is known about what cues are important from the dog’s perspective. Dogs communicate much more with their bodies (e.g., play bow, Bekoff 1977; Byosiere et al. 2016; Horowitz 2009), and there is evidence that full body motion is significant for dogs (Delanoeije et al. 2020; Eatherington et al. 2019; Ishikawa et al. 2018; Kovács et al. 2016). Even though humans still communicate a lot of emotion information with their bodies (e.g., Martinez et al. 2016), faces with emotional cues are more important or informative for humans than for dogs (e.g., Correia-Caeiro et al. 2021). Thus, perhaps not surprisingly when presented with whole human or dog figures, dogs attend more to emotion cues from bodies than from faces whereas humans attend more to faces than bodies (Correia-Caeiro et al. 2021), suggesting a marked difference in how both species perceive emotion cues.

## Summary and general conclusions

In this critical review of the concepts and methodologies commonly used when investigating the visual perception of emotion cues in dogs, we aimed to briefly synthesise relevant results while critically evaluating methodologies, in terms of their ability to make conceptual contributions to the field. In addition, we present frequent challenges in the literature and suggest crucial points for consideration and recommendations and outstanding questions for future research (Table 2). We began by justifying why the domestic dog is an excellent model to investigate the visual perception of emotion cues, given its phylogenetic and ontogenetic adaptation to the human environment (“Why is the dog a good model for research on the perception of emotion cues?”). We answer our second question concerning what is known about the mechanisms of perceiving emotion cues in dogs (“What is known about how dogs visually perceive emotion cues?”), by focusing on studies that demonstrated a set of refined skills for visual perception of emotion cues in dogs. It is clear that dogs have the ability to discriminate and respond to facial expressions both in humans and dogs, but some inconsistent results demand further research into this. Comparative research between humans and dogs has been revealing both similarities (e.g., importance of emotion cues: Correia-Caeiro et al. 2021; Thompkins et al. 2021) and differences (e.g., how facial expressions are perceived, Correia-Caeiro et al. 2020). However, both for face and body perception, it was also clear that more comparative studies are needed (i.e., four-way studies with human and dog participants exposed to human and dog stimuli, e.g.,Correia-Caeiro et al. 2020, 2021). Furthermore, the notable gap in empirical studies in how dogs process body cues both in conspecifics and in humans reveals deep anthropomorphic and anthropocentric biases. In “What methodologies have been used to assess the perception of emotion cues in dogs?”, we answer our third question concerning how emotion cue perception has been measured in dogs, by critiquing methodologies commonly used to collect and analyse each of them (grouped by neurobiological, physiological, and cognitive and behavioural indicators). Finally, in “What are the limitations and challenges to investigate the perception of emotion cues in dogs?”, we detail important limitations and challenges associated with measuring the perception of emotion cues in dogs, and list points to consider and recommendations for future studies. Small/easy to implement adjustments (Table 2) based on our critique in this section, in most instances have the potential to increase robustness, reliability, and validity in future studies. The research area of emotion cue processing in dogs could strengthen its methodological approach if it more often acknowledged and then justified the balance between the ecological and functional relevance of the experimental design with the validity, reliability, and objectivity of the methods used. There is a need for a clearer conceptual foundation, where consideration should be given to the underlying operational definitions for each hypothesis investigated (Correia-Caeiro 2017). Widely varied and cutting-edge equipment, methods, and techniques are already applied in this area (or at least in the more broader cognition/perception areas), such as DogFACS, fMRI, EEG, fNIRS, fixed and mobile eye-tracking, thermal imaging, and physiological monitors, which allow the objective measure of the dogs’ behavioural, physiological, and neurological responses when viewing emotion cues. Nonetheless, these cutting-edge techniques should be applied alongside careful consideration of individual differences (i.e., by using larger sample sizes), and experimental and stimulus design to ensure inferences are valid.

There are still many challenges to overcome in future studies. First, we need to shift the focus of canine research away from an anthropocentric perspective as evidenced by the face-centrism given to social interactions, to a more lupomorphic one, for example, by including body emotion stimuli (Correia-Caeiro et al. 2021). Second, we need to avoid anthropomorphic practices, in which we (often unconsciously) limit studies in dogs to stereotypical “human emotions”, and consider more consistent emotion categories with biologically-based definitions (Bremhorst et al. 2019; Correia-Caeiro et al. 2017) (also see “A brief summary of the nature of emotion and its perceptual processes” for more about the emotion definition debate). Third, although both dogs and humans make extensive use of their vision to perceive emotion cues, it must be appreciated that other sensorial inputs and multimodality are also important, and the interaction between vision and these other modalities needs to be considered if we wish to truly understand how another species perceives emotion cues. So long as we acknowledge these constraints and potential biases and carefully consider their relevance to our research questions, the dog will remain a unique species for providing insights into the common evolutionary basis of emotion cues perception and its origins in humans and other animals.

## Supplementary Information

Below is the link to the electronic supplementary material.

Supplementary file1 (DOCX 62 KB)
