# Supplementary material for: Visual perception of emotion cues in dogs: a critical review of methodologies
Source: Anim Cogn. 2023 Mar 4;26(3):727–54. doi: 10.1007/s10071-023-01762-5 (PMC10066124; doi:10.1007/s10071-023-01762-5)
Supplement: Supplementary file 1 — Supplementary file1 (DOCX 62 KB) [file 10071_2023_1762_MOESM1_ESM.docx]

**SUPPLEMENTARY TEXT FOR:**

**Visual perception of emotion cues in dogs - A critical review of methodologies**

Catia Correia-Caeiro,^1,2,3,4^* Kun Guo,^1^ Daniel S. Mills^2^

^1^School of Psychology, University of Lincoln, Brayford Pool, Lincoln, LN6 7TS, UK.

^2^Department of Life Sciences, University of Lincoln, Lincoln, LN6 7DL, UK.

^3^Primate Research Institute, Kyoto University, Inuyama, 484-8506, Japan. (current address)

^4^Center for the Evolutionary Origins of Human Behavior, Kyoto University, Inuyama, 484-8506, Japan. (current address)

*Corresponding author: [catia_caeiro@hotmail.com](mailto:catia_caeiro@hotmail.com)

**S1: The importance of the visual channel in dogs:**

Emotion cues are salient stimuli that can be detected from the environment through different sensory channels, namely somatosensory, gustatory, olfactory, visual and auditory. In mammals, the emotion-triggering stimuli from the environment perceived through different sensory modalities are integrated in the amygdala and then projected to emotion-category-specific brain regions, which in turn generate specific emotion responses in the individual (McDonald, 2020). Whilst socially salient environmental stimuli are often multimodal, some sensory channels seem to be more relevant for perception of emotion cues than others, and attention may be focused on a specific modality according to circumstances. Somatosensory and gustatory inputs are both very limited as they require physical touch and can only convey either one-on-one messages (e.g., licking) or message quality (e.g., rougher touch vs lighter touch). Auditory exchanges can be used flexibly, at any distance between individuals and/or in visually busy environments, and can be perceived even without a particular focus on the sender. Olfactory cues (e.g., object scent rubbing) are usually longer lasting, and while they can be perceived in real-time or with a delay, they cannot be easily stopped or hidden (i.e., because scents take time to dissipate within the environment), which makes them inflexible, and less practical for complex exchanges (i.e., its long-lasting nature prevents a turn taking production because they can potentially mask each other)). In contrast, visual cues are usually short, can be rapidly collected and assimilated, and allow turn taking, to generate a large amount of information in real time (e.g., sequential and simultaneous body postures and facial movements), however they depend on an obstacle free environment and/or proximity (Liebal et al., 2014; Marler, 1967; Partan & Marler, 1999); therefore it is ideal for relative close social interactions. In this review, we focus on visual perception, as it appears to be generally the most informatively rich channel for exchange of emotion cues between individuals, that has also been most extensively studied; although we acknowledge this might reflect an anthropocentric bias.

Although dogs are macrosmatic (Hepper & Wells, 2005; Walker et al., 2006), and smell may carry certain emotion information (Donovan, 1969), it is clear that in many settings, dogs prioritise other sensory channels according to their potential value to the task at hand, e.g., verbal cues in a detour task (Pongrácz et al., 2004) or visual cues for food detection (Szetei et al., 2003). Dog's vision is not as poor as once thought (Barber et al., 2020; Miller & Murphy, 1995), and within dog-dog social interactions, visual environment is potentially important. For instance, studies have shown that lateralised tail wagging is both displayed as a response to emotion triggers (e.g., snake: Quaranta et al., 2007; food: (Travain et al., 2016) and perceived as a relevant visual signal carrying emotion information (Siniscalchi et al., 2013). The same may also be true for head turning in dogs. For example, when dogs see a potentially negative stimuli they turn their head to the left (Siniscalchi et al., 2010). So, dogs, not only seem to perceive different types of visual emotion stimuli in conspecifics, but they also respond with overt visual behaviours, suggesting that the visual domain is important for dogs both for acquiring and responding to information. Indeed, outside of the emotion domain, visual communication has been shown to be preferred to the verbal channel in dogs for simple tasks, such as in the exchange of declarative information for the direction to sit or lie down (D’Aniello et al., 2016; Scandurra et al., 2017).

**Table S1.** Summary of this section studies comparing dog's perception of behaviour cues in humans vs. dogs.

| **Aim** | **Reference** | **Method used** | **Differences when looking at:** | | **Common to human and dog behaviour** |
| --- | --- | --- | --- | --- | --- |
|  |  |  | **Human behaviour** | **Dog behaviour** |  |
| Social referencing | (Merola et al., 2012) | Exposure to threatening/ambiguous object | Gazed at humans to look for information in ambiguous situations; mirrored human's approach-avoidance behaviour. | - | - |
| Facial expression discrimination | (Nagasawa et al., 2011) | Two-choice discrimination task of pictures | Discriminated between smiling vs neutral expressions | - | - |
|  | (Buttelmann & Tomasello, 2013) | Two-choice discrimination task of a box with hidden contents after a human demonstrator reacted to the box | All dogs discriminated between happy vs. disgusted expressions; some dogs discriminated happy vs. neutral expressions | - | - |
|  | (Müller et al., 2015) | Two-choice discrimination task of pictures on a touchscreen | Discriminated between happy vs. angry expressions | - | - |
| Facial expression gaze bias | (Racca et al., 2012) | Exposure to pictures | Presented left gaze bias for neutral expressions, but no bias for positive expressions | Presented right gaze bias for positive expressions, but no bias for neutral expressions | Presented left gaze bias for negative expressions |
| Facial expression perception | (Somppi et al., 2016) | Eye-tracking during exposure to pictures | Attended more to threatening expressions | Avoided threatening expressions | Gazed more at eyes > midface > mouth |
|  | (Barber et al., 2016) | Eye-tracking during exposure to pictures | Presented left gaze bias for neutral, happy angry and sad expressions; attended more to forehead in positive expressions and eyes and mouth in negative expressions | - | - |
|  | (Correia-Caeiro et al., 2020) | Eye-tracking during exposure to videos | Attended more to the frontalis, eyes, nose, and cheeks during facial expressions | Attended more to the ears, mouth, and eyes | Emotion and species did not change gaze |
| Bodily expression perception | (Correia-Caeiro et al., 2021) | Eye-tracking during exposure to videos | Attended more to the human than the dog body | Attended more to the dog than the human head | Attended more to the body than the head in both species |
| Lateralised tail wagging discrimination | (Siniscalchi et al., 2013) | Exposure to videos | - | Produced differential behavioural and physiological responses to left vs. right tail wag | - |

**S2: Implicit vs explicit measures of emotion cues perception:**

The majority of studies in the area of human emotion (whether investigating perceptual, expression or experience of emotions) rely heavily on self-report and other explicit processes. For example, despite agreement that emotion experiences (and consequently, the expression and perception of its potentially associated cues) are highly subjective, it is common practice to design stimuli representing varied emotional cues (e.g., facial expressions) based solely on common sense of how these cues look like (e.g., a generic smiling human face represents a “happy face”, is perceived as a “happy face” and necessarily means the subject was “happy” when that “happy face” was recorded). However, not only this is not a scientifically rigorous approach, but importantly the identification of emotions are usually not an explicit process (Dimberg et al., 2000; Matsuda et al., 2013; Niedenthal, 1990; Whalen et al., 1998). Indeed, implicit processes might have advantages and these are commonly used in studies involving non-verbal human infants.

Perceptual processes for emotion cues are independent of language (Sauter, 2018) or the ability to linguistically label them (Lang et al., 2000), and thus, language is not the only, nor even the more reliable/valid measure of emotion cues perception (Chan, 2009; Nosek, 2007). More implicit measures are likely a better alternative (especially) for non-human animals to investigate emotion cues (but see other studies for examples of explicit paradigms of dogs (Savalli et al., 2009, 2013) and apes (Savage-Rumbaugh & Rumbaugh, 1978) using lexigrams, or apes (Bonvillian & Patterson, 1993; Jensvold, 2014) using sign language). Even when subliminal (i.e., below an awareness threshold), the perception of valenced stimuli (e.g., a facial expression) directly changes or biases cognitive processing, including decision-making, judgment, memory recall, etc. (Almeida et al., 2013; Ong et al., 2014; Prochnow et al., 2013; Yang et al., 2011), and these can be assessed objectively without language.

Studies on human infants illustrate well a case whereby alternative experimental paradigms to self-report have been developed and extensively validated (Colombo et al., 2012). Identical paradigms, with species-specific modifications (e.g., functionally equivalent experimental setups, additional controls), should thus be valid for dogs. Examples of widely used paradigms to study emotion cues perception in human infants that were successfully adapted to dogs include visual preferences (Kotsoni et al., 2001; Nagasawa et al., 2011) and eye-tracking (Hoehl, 2014; Somppi et al., 2012).

**References:**

Almeida, J., Pajtas, P. E., Mahon, B. Z., Nakayama, K., & Caramazza, A. (2013). Affect of the unconscious: Visually suppressed angry faces modulate our decisions. *Cognitive, Affective, & Behavioral Neuroscience*, *13*(1), 94–101. https://doi.org/10.3758/s13415-012-0133-7

Barber, A. L. A., Mills, D. S., Montealegre-Z, F., Ratcliffe, V. F., Guo, K., & Wilkinson, A. (2020). Functional Performance of the Visual System in Dogs and Humans: A Comparative Perspective. *Comparative Cognition & Behavior Reviews*, *15*, 44.

Barber, A. L. A., Randi, D., Müller, C. A., & Huber, L. (2016). The Processing of Human Emotional Faces by Pet and Lab Dogs: Evidence for Lateralization and Experience Effects. *PLOS ONE*, *11*(4), e0152393. https://doi.org/10.1371/journal.pone.0152393

Bonvillian, J. D., & Patterson, F. G. P. (1993). Early sign language acquisition in children and gorillas: Vocabulary content and sign iconicity. *First Language*, *13*(39), 315–338. https://doi.org/10.1177/014272379301303903

Buttelmann, D., & Tomasello, M. (2013). Can domestic dogs (Canis familiaris) use referential emotional expressions to locate hidden food? *Animal Cognition*, *16*(1), 137–145. https://doi.org/10.1007/s10071-012-0560-4

Chan, D. (2009). So why ask me? Are self-report data really that bad? In C. E. Lance & R. J. Vandenberg (Eds.), *Statistical and Methodological Myths and Urban Legends: Doctrine, Verity and Fable in the Organizational and Social Sciences* (pp. 309–336). Taylor & Francis.

Colombo, J., Brez, C. C., & Curtindale, L. M. (2012). Infant Perception and Cognition. In I. Weiner (Ed.), *Handbook of Psychology, Second Edition*. John Wiley & Sons, Inc. https://doi.org/10.1002/9781118133880.hop206003

Correia-Caeiro, C., Guo, K., & Mills, D. (2021). Bodily emotional expressions are a primary source of information for dogs, but not for humans. *Animal Cognition*. https://doi.org/10.1007/s10071-021-01471-x

Correia-Caeiro, C., Guo, K., & Mills, D. S. (2020). Perception of dynamic facial expressions of emotion between dogs and humans. *Animal Cognition*, *23*(3), 465–476. https://doi.org/10.1007/s10071-020-01348-5

D’Aniello, B., Scandurra, A., Alterisio, A., Valsecchi, P., & Prato-Previde, E. (2016). The importance of gestural communication: A study of human–dog communication using incongruent information. *Animal Cognition*, *19*(6), 1231–1235. https://doi.org/10.1007/s10071-016-1010-5

Dimberg, U., Thunberg, M., & Elmehed, K. (2000). Unconscious facial reactions to emotional facial expressions. *Psychological Science*, *11*(1), 86–89.

Donovan, C. A. (1969). Canine anal glands and chemical signals (pheromones). *Journal of the American Veterinary Medical Association*, *155*(12), 1995–1996.

Hepper, P. G., & Wells, D. L. (2005). How Many Footsteps Do Dogs Need to Determine the Direction of an Odour Trail? *Chemical Senses*, *30*(4), 291–298. https://doi.org/10.1093/chemse/bji023

Hoehl, S. (2014). Emotion Processing in Infancy. *Children and Emotion*, *26*, 1–12. https://doi.org/10.1159/000354346

Jensvold, M. L. (2014). Experimental Conversations: Sign Language Studies with Chimpanzees. In M. Pina & N. Gontier (Eds.), *The Evolution of Social Communication in Primates: A Multidisciplinary Approach* (pp. 63–82). Springer International Publishing. https://doi.org/10.1007/978-3-319-02669-5_4

Kotsoni, E., de Haan, M., & Johnson, M. H. (2001). Categorical Perception of Facial Expressions by 7-Month-Old Infants. *Perception*, *30*(9), 1115–1125. https://doi.org/10.1068/p3155

Lang, P. J., Davis, M., & Öhman, A. (2000). Fear and anxiety: Animal models and human cognitive psychophysiology. *Journal of Affective Disorders*, *61*(3), 137–159.

Liebal, K., Waller, B. M., Slocombe, K. E., & Burrows, A. M. (2014). *Primate Communication: A Multimodal Approach*. Cambridge University Press.

Marler, P. (1967). Animal Communication Signals: We are beginning to understand how the structure of animal signals relates to the function they serve. *Science*, *157*(3790), 769–774. https://doi.org/10.1126/science.157.3790.769

Matsuda, Y.-T., Fujimura, T., Katahira, K., Okada, M., Ueno, K., Cheng, K., & Okanoya, K. (2013). The implicit processing of categorical and dimensional strategies: An fMRI study of facial emotion perception. *Frontiers in Human Neuroscience*, *7*. https://doi.org/10.3389/fnhum.2013.00551

McDonald, A. J. (2020). Chapter 1 - Functional neuroanatomy of the basolateral amygdala: Neurons, neurotransmitters, and circuits. In J. H. Urban & J. A. Rosenkranz (Eds.), *Handbook of Behavioral Neuroscience* (Vol. 26, pp. 1–38). Elsevier. https://doi.org/10.1016/B978-0-12-815134-1.00001-5

Merola, I., Prato-Previde, E., & Marshall-Pescini, S. (2012). Social referencing in dog-owner dyads? *Animal Cognition*, *15*(2), 175–185. https://doi.org/10.1007/s10071-011-0443-0

Miller, P. E., & Murphy, C. J. (1995). Vision in dogs. *Journal-American Veterinary Medical Association*, *207*, 1623–1634.

Müller, C. A., Schmitt, K., Barber, A. L. A., & Huber, L. (2015). Dogs Can Discriminate Emotional Expressions of Human Faces. *Current Biology*, *25*(5), 601–605. https://doi.org/10.1016/j.cub.2014.12.055

Nagasawa, M., Murai, K., Mogi, K., & Kikusui, T. (2011). Dogs can discriminate human smiling faces from blank expressions. *Animal Cognition*, *14*(4), 525–533. https://doi.org/10.1007/s10071-011-0386-5

Niedenthal, P. M. (1990). Implicit perception of affective information. *Journal of Experimental Social Psychology*, *26*(6), 505–527. https://doi.org/10.1016/0022-1031(90)90053-O

Nosek, B. A. (2007). Implicit–explicit relations. *Current Directions in Psychological Science*, *16*(2), 65–69.

Ong, H. H., Mullette-Gillman, O. A., Kwok, K., & Lim, J. (2014). Moral judgment modulation by disgust is bi-directionally moderated by individual sensitivity. *Frontiers in Psychology*, *5*. https://doi.org/10.3389/fpsyg.2014.00194

Partan, S., & Marler, P. (1999). Communication Goes Multimodal. *Science*, *283*(5406), 1272–1273.

Pongrácz, P., Miklósi, Á., Timár-Geng, K., & Csányi, V. (2004). Verbal Attention Getting as a Key Factor in Social Learning Between Dog (Canis familiaris) and Human. *Journal of Comparative Psychology*, *118*(4), 375–383. https://doi.org/10.1037/0735-7036.118.4.375

Prochnow, D., Kossack, H., Brunheim, S., Müller, K., Wittsack, H.-J., Markowitsch, H.-J., & Seitz, R. J. (2013). Processing of subliminal facial expressions of emotion: A behavioral and fMRI study. *Social Neuroscience*, *8*(5), 448–461. https://doi.org/10.1080/17470919.2013.812536

Quaranta, A., Siniscalchi, M., & Vallortigara, G. (2007). Asymmetric tail-wagging responses by dogs to different emotive stimuli. *Current Biology*, *17*(6), R199–R201.

Racca, A., Guo, K., Meints, K., & Mills, D. S. (2012). Reading Faces: Differential Lateral Gaze Bias in Processing Canine and Human Facial Expressions in Dogs and 4-Year-Old Children. *PLoS ONE*, *7*(4), e36076. https://doi.org/10.1371/journal.pone.0036076

Sauter, D. A. (2018). Is There a Role for Language in Emotion Perception? *Emotion Review*, *10*(2), 111–115. https://doi.org/10.1177/1754073917693924

Savage-Rumbaugh, E. S., & Rumbaugh, D. M. (1978). Symbolization, language, and chimpanzees: A theoretical reevaluation based on initial language acquisition processes in four young Pan troglodytes. *Brain and Language*, *6*(3), 265–300. https://doi.org/10.1016/0093-934X(78)90063-9

Savalli, C., Brandão, M. M., Domingues, T. S., Honório, M. A., & Ades, C. (2009). Dog-Human communication through a keyboard: Is human attentional state relevant? *Journal of Veterinary Behavior*, *4*(2), 55. https://doi.org/10.1016/j.jveb.2008.09.054

Savalli, C., Resende, B. D. de, & Ades, C. (2013). Are dogs sensitive to the human’s visual perspective and signs of attention when using a keyboard with arbitrary symbols to communicate? *Revista de Etologia*, *2175–3636*. https://doi.org/10.22491/etologia.v12n1-2.art5

Scandurra, A., Alterisio, A., Marinelli, L., Mongillo, P., Semin, G. R., & D’Aniello, B. (2017). Effectiveness of verbal and gestural signals and familiarity with signal-senders on the performance of working dogs. *Applied Animal Behaviour Science*, *191*, 78–83. https://doi.org/10.1016/j.applanim.2017.02.003

Siniscalchi, M., Lusito, R., Vallortigara, G., & Quaranta, A. (2013). Seeing Left- or Right-Asymmetric Tail Wagging Produces Different Emotional Responses in Dogs. *Current Biology*, *23*(22), 2279–2282. https://doi.org/10.1016/j.cub.2013.09.027

Siniscalchi, M., Sasso, R., Pepe, A. M., Vallortigara, G., & Quaranta, A. (2010). Dogs turn left to emotional stimuli. *Behavioural Brain Research*, *208*(2), 516–521. https://doi.org/10.1016/j.bbr.2009.12.042

Somppi, S., Törnqvist, H., Hänninen, L., Krause, C., & Vainio, O. (2012). Dogs do look at images: Eye tracking in canine cognition research. *Animal Cognition*, *15*(2), 163–174. https://doi.org/10.1007/s10071-011-0442-1

Somppi, S., Törnqvist, H., Kujala, M. V., Hänninen, L., Krause, C. M., & Vainio, O. (2016). Dogs evaluate threatening facial expressions by their biological validity–Evidence from gazing patterns. *PloS One*, *11*(1), e0143047.

Szetei, V., Miklósi, á, Topál, J., & Csányi, V. (2003). When dogs seem to lose their nose: An investigation on the use of visual and olfactory cues in communicative context between dog and owner. *Applied Animal Behaviour Science*, *83*(2), 141–152. https://doi.org/10.1016/S0168-1591(03)00114-X

Travain, T., Colombo, E. S., Grandi, L. C., Heinzl, E., Pelosi, A., Prato Previde, E., & Valsecchi, P. (2016). How good is this food? A study on dogs’ emotional responses to a potentially pleasant event using infrared thermography. *Physiology & Behavior*, *159*, 80–87. https://doi.org/10.1016/j.physbeh.2016.03.019

Walker, D. B., Walker, J. C., Cavnar, P. J., Taylor, J. L., Pickel, D. H., Hall, S. B., & Suarez, J. C. (2006). Naturalistic quantification of canine olfactory sensitivity. *Applied Animal Behaviour Science*, *97*(2), 241–254. https://doi.org/10.1016/j.applanim.2005.07.009

Whalen, P. J., Rauch, S. L., Etcoff, N. L., McInerney, S. C., Lee, M. B., & Jenike, M. A. (1998). Masked presentations of emotional facial expressions modulate amygdala activity without explicit knowledge. *Journal of Neuroscience*, *18*(1), 411–418.

Yang, J., Xu, X., Du, X., Shi, C., & Fang, F. (2011). Effects of Unconscious Processing on Implicit Memory for Fearful Faces. *PLOS ONE*, *6*(2), e14641. https://doi.org/10.1371/journal.pone.0014641
